# Omics Studies of Tumor Cells under Microgravity Conditions

**DOI:** 10.3390/ijms25020926

**Published:** 2024-01-11

**Authors:** Jenny Graf, Herbert Schulz, Markus Wehland, Thomas J. Corydon, Jayashree Sahana, Fatima Abdelfattah, Simon L. Wuest, Marcel Egli, Marcus Krüger, Armin Kraus, Petra M. Wise, Manfred Infanger, Daniela Grimm

**Affiliations:** 1 Department of Microgravity and Translational Regenerative Medicine, Otto von Guericke University, 39106 Magdeburg, Germany; jenny.graf@med.ovgu.de (J.G.); herbert.schulz@med.ovgu.de (H.S.); markus.wehland@med.ovgu.de (M.W.); fatima.abdelfattah@med.ovgu.de (F.A.); marcus.krueger@med.ovgu.de (M.K.); wisepetra@gmail.com (P.M.W.); 2 Research Group “Magdeburger Arbeitsgemeinschaft für Forschung unter Raumfahrt- und Schwerelosigkeitsbedingungen” (MARS), Otto von Guericke University, 39106 Magdeburg, Germany; armin.kraus@med.ovgu.de (A.K.); manfred.infanger@med.ovgu.de (M.I.); 3 Department of Biomedicine, Aarhus University, 8000 Aarhus C, Denmark; corydon@biomed.au.dk (T.J.C.); jaysaha@biomed.au.dk (J.S.); 4 Department of Ophthalmology, Aarhus University Hospital, 8200 Aarhus N, Denmark; 5 Space Biology Group, Institute of Medical Engineering, Lucerne University of Applied Sciences and Arts, 6052 Hergiswil, Switzerlandmarcel.egli@hslu.ch (M.E.); 6 National Center for Biomedical Research in Space, Innovation Cluster Space and Aviation (UZH Space Hub), University Zurich, 8006 Zurich, Switzerland; 7 Clinic for Plastic, Aesthetic and Hand Surgery, Medical Faculty, University Hospital Magdeburg, Otto von Guericke University, 39120 Magdeburg, Germany; 8 The Saban Research Institute, Children’s Hospital Los Angeles, University of Southern California, 4650 Sunset Blvd, Los Angeles, CA 90027, USA

**Keywords:** microgravity, weightlessness, space, omics studies, cancer, cancer cells, genomics, transcriptomics, proteomics, metabolomics

## Abstract

Cancer is defined as a group of diseases characterized by abnormal cell growth, expansion, and progression with metastasis. Various signaling pathways are involved in its development. Malignant tumors exhibit a high morbidity and mortality. Cancer research increased our knowledge about some of the underlying mechanisms, but to this day, our understanding of this disease is unclear. High throughput omics technology and bioinformatics were successful in detecting some of the unknown cancer mechanisms. However, novel groundbreaking research and ideas are necessary. A stay in orbit causes biochemical and molecular biological changes in human cancer cells which are first, and above all, due to microgravity (µ*g*). The µ*g*-environment provides conditions that are not reachable on Earth, which allow researchers to focus on signaling pathways controlling cell growth and metastasis. Cancer research in space already demonstrated how cancer cell-exposure to µ*g* influenced several biological processes being involved in cancer. This novel approach has the potential to fight cancer and to develop future cancer strategies. Space research has been shown to impact biological processes in cancer cells like proliferation, apoptosis, cell survival, adhesion, migration, the cytoskeleton, the extracellular matrix, focal adhesion, and growth factors, among others. This concise review focuses on publications related to genetic, transcriptional, epigenetic, proteomic, and metabolomic studies on tumor cells exposed to real space conditions or to simulated µ*g* using simulation devices. We discuss all omics studies investigating different tumor cell types from the brain and hematological system, sarcomas, as well as thyroid, prostate, breast, gynecologic, gastrointestinal, and lung cancers, in order to gain new and innovative ideas for understanding the basic biology of cancer.

## 1. Introduction

A long-term spaceflight strongly impacts human health. Space travelers suffer from various health problems, such as cardiovascular disorders, bone loss, muscle atrophy, dysfunction of the immune system, disturbed wound healing, or pain, among others [1,2,3,4,5,6,7,8,9,10,11,12,13,14,15,16,17,18,19,20,21,22,23,24,25,26,27,28,29].

So far, multiple changes occurring in tissues and on the cellular level have been reported [30,31]. Human cell cultures of benign and malignant cells exposed to real microgravity (r-µ*g*, also called weightlessness) in space showed several morphological and molecular changes [32,33,34,35,36,37,38,39,40,41]. Low differentiated FTC-133 follicular thyroid cancer cells studied in space for 10 days (d) during the Shenzhou-8 space mission showed changes in growth behavior and exhibited two different phenotypes after spaceflight. In spaceflight samples, one part of the cells grew in the form of multicellular spheroids (MCS); the other one remained growing adherently as a monolayer on the bottom of the hardware chamber. Gene arrays and quantitative real-time (rt) PCR studies demonstrated that genes involved in different biological processes were differentially expressed in space [42].

Omics in space is currently a hot topic in space biology and space medicine. One reason for this increased interest is the exploration of outer space. The National Aeronautics and Space Administration (NASA) GeneLab allows open sciences for space research [43]. It comprises the first comprehensive space-related omics database and contains transcriptomics, genomics including metagenomics and epigenomics, proteomics and metabolomics data from space, and µ*g* simulation studies performed on devices designed to create a µ*g*-environment on Earth [44].

This novel information helps to determine the space traveler’s health risks during deep space exploration and supports the detection of genes and proteins in cancer cells exposed to µ*g*, which might be involved in signal transduction pathways and may serve as possible future targets for cancer treatment. Cancer is a burden on mankind. In 2022, 1,918,030 new cancer cases and 609,360 cancer deaths were predicted to occur in the United States [45], and, according to the GLOBOCAN data base worldwide, an estimated 19.3 million new cancer cases (18.1 million excluding non-melanoma skin cancer) and almost 10.0 million cancer deaths (9.9 million excluding non-melanoma skin cancer) occurred in 2020 [46]. The number of these cases is increasing and therefore new treatment options need to be developed. Cancer research experiments conducted in orbit have demonstrated how exposure to µ*g* influences biological processes that may be relevant to cancer [47,48].

The principal aims of this concise review are (1) to explore the influence of µ*g* on cancer cell biology, focusing on tumor cells cultured in space together with experiments carried out using models conducted with ground-based µ*g*-simulators and (2) to review the available literature reporting in vitro cancer studies using omics investigations in r-µ*g* and simulated (s-)µ*g*. This knowledge will help to develop new concepts in the field of cancer research. Furthermore, spaceflight findings are also beneficial for translational regenerative medicine on Earth.

## 2. Microgravity Platforms

**Figure 1:**
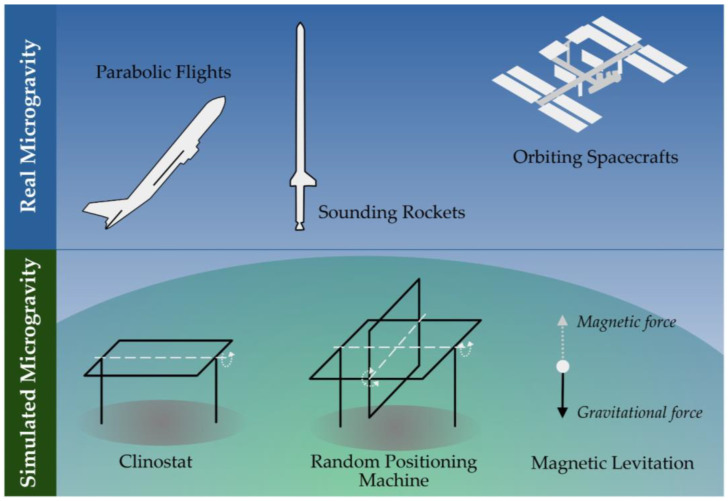
(**Top**)**:** Representations of platforms suitable to perform experiments in real microgravity (parabolic flight aircraft, sounding rocket, and orbiting spacecraft such as the international space station). (**Bottom**)**:** Schematic illustration of clinostat and Random Positioning Machine rotations as well as magnetic levitation where the magnetic force counteracts of the gravitational force.

Establishing a µ*g*-environment essentially means that the objects studied are kept in a free-fall condition [49]. Several research platforms are currently available on which free-fall conditions and, thus, r-µ*g* are provided. In contrast to bringing samples into free fall, µ*g* can also be simulated on ground by counteracting the gravitational force or mathematically nullifying the gravity vector [50,51]. The latter is usually done by distributing the force of gravity equally in space and time by rotating the samples. These different µ*g*-platforms have both their technical advantages and drawbacks. What these platforms for r-µ*g* have in common is that the transition into and from µ*g* happens through a period of hypergravity (elevated gravity). The characteristics of the various platforms (Figure 1) are highlighted in the following paragraphs.

Airplanes performing parabolic flights provide a frequently used µ*g*-research platform. If conducted in large aircrafts (such as the European Airbus A310 Zero G), scientists can interact with their experiments during the flight, which is a major advantage [52]. Thus, there is no need to develop fully automated experimental hardware, hereby reducing the constraints and overall costs of the scientific instrumentation. This allows for modified commercial and off-the-shelf analytical devices to be flown after minor technical modifications and safety assessments.

**Figure 2:**
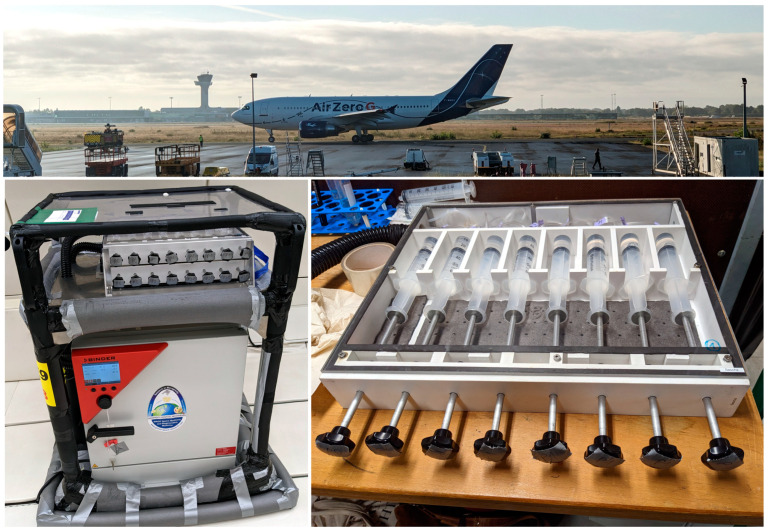
Impressions of the 41st DLR parabolic flight campaign in September 2023, Bordeaux, France. The Airbus A310 Zero G aircraft of Novespace (**top**), the flight rack of the “Thyroid” mission (**bottom left**) and its RNA*later* injection system (**bottom right**) are shown.

During parabolic flights, the aircraft flies along a parabolic trajectory, bringing the samples on board into free-fall. The parabolic maneuver of the Airbus A310 (Novespace, Bordeaux-Merignac, France) (Figure 2), for example, which is in service for the European Space Agency (ESA), starts from a horizontal flight and transits into a steep incline, culminating in a flight angle of roughly 50° after about 20 s. During this initial phase, the experimental payload experiences hypergravity of typically 1.5 to 1.8*g*. Next, the airplane’s thrust is reduced to compensate for the air drag, and the aircraft flows along a parabolic trajectory for around 22 s. This is the actual period during which the payload inside the aircraft experiences µ*g*. The maneuver ends by pulling the plane out of a deep dive back into a horizontal flight. Like in the initial phase, hypergravity of about 1.8*g* also builds up during this final phase. Typically, this maneuver is repeated several times with short pauses between the parabolas. The major drawback of parabolic flights is the relatively short µ*g* period of about 22 s and the moderate µ*g* quality of about 10^−1^*g* [53,54].

More extended µ*g* periods can be achieved by sounding rockets and, depending on the utilized carrier, µ*g* periods of several minutes can be reached. Typical µ*g* periods are around 5 to 6 min, but stronger rockets have been employed as well. Sounding rockets fly the payload beyond the Earth’s atmosphere (apogees reaching often around 260 km but can go up to ca. 700 km). The acceleration is very severe during launch, (approx. 13*g*) and the samples must endure strong vibrations. After the burn-out, the rocket motor is detached from the payload for the subsequent free-fall phase. Furthermore, the payload is stabilized to avoid rotation and tumbling, which could impact the µ*g* quality. The µ*g* period is terminated as the payload hits the upper layer of the Earth’s atmosphere. As the rocket tumbles to the lower atmospheric layers, vibrations get stronger until a parachute opens, allowing the payload to glide down to Earth safely, where it is picked up by a helicopter [55].

Sounding rockets can provide high-quality µ*g* (typically around 10^−4^*g*) for several minutes. However, they also pose the harshest launch conditions for the experiment among all µ*g* platforms. This challenges the experiment and the hardware design, especially for investigating biological samples. Thus, sounding rockets are much more demanding on hardware development than parabolic flights. Therefore, a higher degree of automatization and optimization of hardware size, weight, and power consumption is required [55].

Even longer µ*g* periods can be provided by orbiting platforms, such as unmanned return satellites or crewed space stations. Experiments on return satellites are integrated into the satellite before launch and recovered after successful landing [56]. Like sounding rocket flights, return satellites are demanding on hardware development, as the experiments need to run fully automatized and are on a tight budget of allowable size, weight, and energy consumption. Furthermore, the temperature needs to be stabilized to shield the sample from the extreme temperature fluctuation in orbit. Under the Russian BION space program, for example, several return satellites were launched to stay in low orbit for 5 to 22 d [57].

Experiments on space stations (such as the International Space Station, ISS) are often integrated into an existing facility on the station. Experiments are sent to the station and returned as part of a resupply mission. Space stations have the advantage of allowing astronaut interventions to deploy, handle the experiment, or even troubleshoot in cases of unexpected malfunctions. However, as crew time is limited, experiments still need a high degree of automatization or remote-control capabilities. As space stations provide a habitable environment (room temperature and a terrestrial atmosphere) and facilities with standardized interfaces, the hardware requirements are less demanding than those of other orbiting platforms. Furthermore, the ISS provides cold stowage facilities (e.g., GLACIER or MELFI), allowing the storage of samples until deployment or keeping fixed samples until download. Experiments on return satellites and space stations are both typically launched into orbit on large rocket systems. On these systems, the physical stress on the samples during launch is moderate compared to sounding rocket flights (max. 4 to 6*g*). Some launchers allow warm, cold, frozen, or powered sample uploads. For both platforms, the possibility of launch delays or scraps are eminent and generates problems, especially for life science investigations. Because the timeline of biological experiments is essential, a delay in the launch can interfere with the experiment outcome. A further caveat in using these platforms is that samples are also exposed to space radiation. Therefore, to control the influence of radiation, some experiments use in-flight 1*g* centrifuges to compare microgravity samples to normal (terrestrial) gravity control samples [58,59].

**Figure 3:**
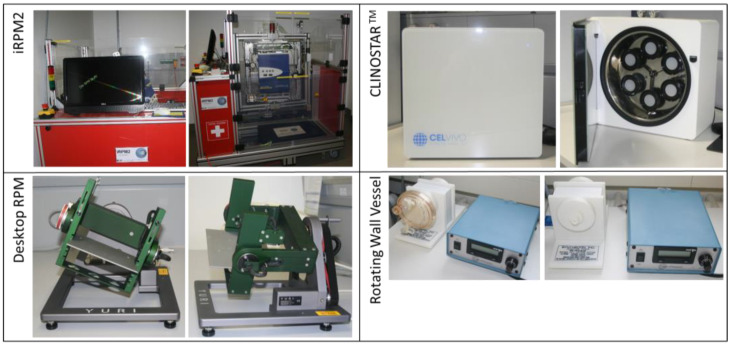
Ground-based s-µ*g* facilities: (**Top left**): The Incubator Random Positioning Machine (iRPM) developed by the ‘Fachhochschule Nordwestschweiz’ (FHNW) and the ‘Eidgenössische Technische Hochschule’ (ETH) Zurich, Switzerland [71]. (**Top right**): the ClinoStar CO_2_ incubator with integrated clinostat purchased from CelVivo ApS, Odense, Denmark. (**Bottom left**): The desk-top Random Positioning Machine provided by Yuri GmbH, Meckenbeuren, Germany. (**Bottom right**): The NASA-developed Rotating Wall Vessel purchased from Synthecon, Houston, TX, USA.

Due to the costly nature and long turnaround times of space-flown experiments, multiple ground-based µ*g*-simulation devices are in use (Figure 3) [50,51]. It is important to state that none of these µ*g*-simulation devices create a true µ*g*-environment in a physical sense. However, they are valuable tools for pre- and post-flight experiments and ground tests during hardware development. Despite the fact that some space-flown experiments were reliably reproduced on µ*g*-simulation devices, results from s-µ*g* should always be discussed critically in the context of confounding factors that could jeopardize a true µ*g* effect [60,61].

The most common s-µ*g* approach is via rotation (Figure 3). Due to the small size of the devices and ease of use, they have become common in dedicated labs. For life science experiments, it is assumed that organisms (such as cells) need a minimum time to adapt to the Earth’s gravity vector. By rotating the sample faster than the process studied, the sample experiences a µ*g*-like condition. However, rotation should not be too fast either, as this will introduce stresses not related to µ*g* [60,62].

Clinostats [63,64] and the related rotating wall vessels (RWVs) [65,66] rotate the samples around a single horizontal axis. RWVs are most often used on suspended samples, and the rotation speed is adjusted, so that the samples stay in suspension and never sediment. Culture chambers for RWVs typically have a larger diameter and volume but rotate more slowly than clinostats. In contrast, clinostats are used on suspended and adherent samples. For suspended samples, the rotation should also avoid sedimentation such that the sample experiences continuous “free-fall”. Some fast-rotating clinostats use rotation speeds up to 100 rpm and culture chambers with small diameters (e.g., 10 mL serological pipettes) to reduce centrifugal forces. Both systems have the advantage that, after an initial start-up phase, the medium flow inside the culture chamber becomes minimal, avoiding shear stresses on the samples [67]. However, the caveat is that they only work well on small (max. millimeter-sized) samples. Therefore, the Random Positioning Machine (RPM) [60,62] has been developed, which provides a larger experiment volume. RPMs consist of a gimbal-mounted platform rotating the samples continuously around two axes with random speed and direction. Due to the low rotation speed (typically around 10 rpm), centrifugal forces are in the order of magnitude of 10^−1^*g*, even several centimeters away from the center of rotation [60]. Therefore, RPMs are predetermined for larger samples, such as plants or small organs. However, due to the interlinked rotation around two axes, complex fluid motion patterns are introduced in medium-filled flasks, which expose the samples to fluid shearing forces [68,69,70].

As an alternative to rotating systems, magnetic levitation uses strong magnetic fields to counterbalance gravity to keep samples in suspension [72,73,74,75]. While the downward pull of gravity depends on the density of the sample and the Earth’s gravity, the upward pull depends on the magnetic susceptibility of the sample and the magnet used (product of the field strength and the field gradient). Magnetic levitation has the advantage that the levitation condition is met for sample sizes from a few centimeters and down to molecular dimensions. However, as levitation depends on the ratio of the sample’s density to the sample’s magnetic susceptibility, magnetic levitation works exceptionally well with homogenous materials. Mixed samples, such as suspensions or biological cells, are thus more troublesome to levitate. Furthermore, the effect of the strong magnetic fields (15–17 T) should be considered during the study design and data interpretation [76,77]. Finally, due to the specialized facility required, availability has been limited, leading to a reduced publication output of magnetically levitated life science experiments.

In conclusion, r-µ*g* and s-µ*g* platforms have been valuable resources for numerous experiments. Each platform type has technical, financial, and organizational advantages and drawbacks. Combining the results from the different µ*g* platforms allows us to acquire a more complete picture respecting the different time scales of molecular processes and mechanisms.

## 3. Results for Omics Studies in Tumor Cells Exposed to Microgravity

### 3.1. Brain Tumors

A brain tumor is defined as the abnormal growth of cells in or near the brain. Hence, brain tumors can begin in the brain tissue (often referred to as primary brain tumors) or near the brain tissue, including the pituitary gland, nerves, the pineal gland, and the membranes covering the surface of the brain. In some cases, cancer spreads to the brain from other parts of the body. By nature, these tumors are called secondary brain tumors or metastatic brain tumors. There are about 130 different types of brain and central nervous system (CNS) tumors, all ranging from malignant to benign, and from extremely rare to relatively common. The four most common brain tumors are metastatic, meningioma, glioblastoma, and astrocytoma. The exact incidence rates of these tumors are not known. However, the American Brain Tumor Association (www.abta.org, accessed on 6 October 2023)/the American Association of Neurological Surgeons (www.aans.org, accessed on 6 October 2023) estimates between 200,000–300,000 metastatic brain tumors, 42,000 meningioma, 15,000 glioblastomas, and 15,000 astrocytomas are diagnosed each year in the United States. Even though current treatment strategies have improved the survival rate for a cancerous brain (or CNS) tumor, these figures clearly state that brain tumors are a serious health concern. Importantly, for some of the brain tumors, e.g., glioblastoma, survival of patients remains poor [78]. Deeper understanding about the mechanisms by which the space environment effects tumor biology may hopefully be translated into the development of novel treatment options including target drug therapy.

The mechanism of spaceflight-induced effects on cancer cells have been studied extensively during recent decades [47,79] and, in particular, reviewed by Marfia et al. [80]. Here, we focused on genomics, transcriptomics, metabolomics, and proteomics data obtained from human brain tumor cells subjected to r-µ*g* or s-µ*g*. However, to our knowledge, only a limited number of studies involving these topics have been published.

Glioblastoma is the most common primary brain tumor in adults [81]. Collectively, recent studies demonstrated that apoptosis in cultured glia cells, as well as inhibition of proliferation and enhanced sensitivity toward chemicals of glioblastoma cells were induced by µ*g* [82,83]. To investigate the evident effects of µ*g* on glioblastoma cells and how reduced gravity might alter invasion and migration potentials, Shi and colleagues subjected human glioblastoma U87 cells to s-µ*g* using a 2D-clionostat [84]. Importantly, s-µ*g* stimulation significantly reduced the migration and invasion potentials, decreased thapsigargin-induced store-operated calcium entry (SOCE), and downregulated the expression of ORAI1 protein in U87 cells. To further dissect the mechanism behind the observed results, inhibition of SOCE by stromal interaction molecule 1 (STIM1) or 2-aminoethoxydiphenyl borate (2-APB) was pursued. In both cases inhibition of SOCE in the U87 cells mirrored the effects of s-µ*g*. As overexpression of *ORAI1* dramatically reversed the effect of s-µ*g*, the authors suggested that s-µ*g* conditions inhibited the migration and inversion potentials of U87 cells by downregulating the expression of *ORAI1* [84].

Using a novel 3D-bioprinted vascularized glioblastoma-on-a-chip biological platform, Silvani et al. have further advanced our knowledge of how A-172 glioblastoma cells in general react to the exposure to s-µ*g*, and specifically explored molecular targets involved in adhesion processes able to respond to the mechanical unloading condition [85,86]. Of special interest is that the system can recapitulate the compartmentalized brain tumor microenvironment by selectively mimicking, e.g., physiological shear stress and cell–cell mechanical interaction [85]. To investigate the activated mechanosensing pathways involved in cellular adaptation to reduced gravity, the cells were exposed to 24 h of s-µ*g* conditions. The results indicated that glioblastoma cells are susceptible when the gravitational vector is interrupted, substantiating the impact that µ*g* has on cancer cell functionality. Notably, Silvani et al. observed an inactivation of the yes-associated protein 1 (YAP1) in glioblastoma cells, suggesting that this protein represents a molecular entry point and that mechanical unloading inhibited proliferation of the glioblastoma cells due to the inactivation of YAP1 convoyed by the absence of anchoring points to the actin cytoskeleton resulting in reduced cellular structural stability [86].

Taken together, the study provides support for the application of the proposed biological platform for advancing space mechanobiology research, thus also highlighting perspectives and strategies for developing the next generation of brain cancer molecular therapies, including targeted drug delivery strategies [86].

Neural stem cells (NSCs) are the basis for the regeneration of the CNS cell populations. Investigations into how weightlessness influences NSCs in health and disease states, offer an important instrument for the potential alleviation of the specific mechanisms leading to, for example, neurological disorders. Hence, previous research studies have investigated the effects of s-µ*g* on NSCs. One important finding from these studies was that murine cerebellar NSCs react to s-µ*g* by the arrest of their cell cycle in the S-phase with an associated increase in apoptosis [87].

To avoid the use of s-µ*g*, a recent study by Shaka et al. set out to investigate whether r-µ*g* induced proliferation abnormalities of cytokinesis. In addition, the authors took a closer look at proliferation abnormalities in single cell division. Using time-lapse microscopy analysis, the study found frequent occurrence of abnormal cell division (ACD), including incomplete cell division (ICD), where cytokinesis is not successfully completed, and multi-daughter cell division (MDCD) of NSCs subjected to r-µ*g* compared to ground controls (1*g*) [88]. The study not only supports previous data obtained in s-µ*g*, but also shows an increased appearance of more than two daughter cells, as would the characteristic for a single cell division after cytokinesis be. To investigate whether these observed changes occurred in the cells directly during spaceflight, the authors focused on molecules that would have been secreted by the NSCs under the influence of µ*g*. Interestingly, the secretome produced similar rates of ICD, suggesting that secreted molecules rather than mechanistical forces are responsible for the observed effects [88]. Finally, the authors asked the question whether the space-produced secretome would induce ICD and MDCD in naïve NSCs that have never been subjected to r-µ*g*. A clear tendency towards increased ICD and MDCD was observed in naïve NSCs incubated with space-produced secretome compared to ground controls [88].

These findings may thus provide novel insight into the impact of µ*g*-induced health implications of space travelers. The authors also suggested that the provided data may deliver important knowledge for future developments of precautionary or curative actions for a successful long-term r-µ*g*-exposure. However, further studies are needed to investigate, for example, the composition of the space-produced secretome.

### 3.2. Hematological Malignancies

Hematological disorders are a type of malignancy that affects the bone marrow and blood cells with three primary varieties: leukemia, lymphoma, and myeloma. Leukemia occurs when normal white blood cells are produced too quickly, hindering their ability to fight against infections and impacting the bone marrow’s capacity to produce red blood cells and platelets. Lymphomas affect the lymphatic system, which enables the body to eliminate excess fluid and create immune cells. The multiple myeloma is a type of blood neoplasm that mainly impacts plasma cells and leaves the immune system weak and susceptible to infection by preserving the body’s average production of antibodies [89]. Blood malignancies claim the lives of over 720,000 people annually worldwide, accounting for more than 7% of all cancer-related deaths. Due to the poor prognosis of patients with hematological malignancies, there is an increased need for cancer research that could lead to the development of new drugs to alleviate symptoms [45,90,91].

Over the past four decades, research has shown that exposure to µ*g* can alter biological processes. µ*g* has significantly impacted tumor cells, affecting cellular proliferation, apoptosis, invasion, migration, and gene expression. Stem and cancer stem cells may also be similarly involved [92]. Moreover, it has been observed that µ*g* can change the way chemotherapy drugs work. However, there have been limited r-µ*g* studies or animal experiments in space, while most research was conducted on cell culture lines under s-µ*g* conditions [93,94]. Tumor diseases remain the most significant global challenges and, despite scientific advancements in medicine, no conclusive methods have been discovered to prevent tumor development or guide its treatment. Therefore, a new therapeutic approach might be developed using µ*g* settings. Numerous investigations were carried out to examine the theory that µ*g* has anti-cancer potential by suppressing cell growth [82,95,96]. Studies of tumor cells using OMIC techniques are crucial because they provide insights into the molecular mechanisms that drive tumor cell plasticity and heterogeneity. Understanding these mechanisms is essential for developing precision medications that target malignancies more effectively. Tumor heterogeneity, which includes genetic, epigenetic, phenotypic, and functional changes, is a critical factor in the progression of cancers, as it promotes resistance to treatment and accelerates metastasis [97,98].

The researchers used biological equipment, databases, and structures to analyze around 8000 biochemical pathways using global gene expression data from human cells in µ*g* that have already been published. They discovered nearly 100 new pathways, including downregulating the NFκB pathway via Notch1 signaling, a new path for reduced immunity in µ*g*. They also found that this pathway could lead to cancer and increase the drug response to cancer in µ*g*. The study identified numerous other pathways with statistical confidence for each dataset [99]. A recent publication showed that cancer cells respond differently to treatment under µ*g* conditions. The authors suggested that s-µ*g* could create new space and terrestrial medicine immunotherapies. Specifically, leukemic cancer cells treated with daunorubicin showed increased chemotactic migration after exposure to s-µ*g* for longer than ordinary gravity. The study also found that K562 cells, which are derived from patients with chronic myelogenous (myeloid) leukemia (CML), produced more reactive oxygen species expression (ROS) than HL60 cells, which were derived from patients with acute myeloid leukemia (AML) [100].

During a spaceflight study, researchers examined two cell lines derived from Burkitt lymphoma—one positive for Epstein–Barr virus (RAJI cells) and the other negative (BJAB cells). They evaluated the expression of the anti-apoptotic protein BHRF1 (or vBcl-2) to investigate the effects of EBV on apoptosis in both cell lines. The results indicated that the EBV-negative cell line experienced a decreased viability and increased apoptosis, while EBV infection offered protection against apoptosis. Furthermore, µ*g* reduced DNA repair in Raji cells, which exhibited higher ROS and reduced ataxia-telangiectasia, mutated (ATM) expression compared to their non-EBV-infected BJAB cell line. EBV and modeled microgravity also appeared to impact the nuclear localization of ATM, potentially further decreasing its effectiveness [101].

Another study compared the lymphocyte cells from a healthy person with cells from the U937 cell line. RPM exposure resulted in apoptosis in lymphocytes by 5-lipoxygenase-mediated mitochondrial uncoupling and cytochrome c release since its activity fourfold increased within 2 h. In contrast, the U937 cell line did not express an active 5-LOX [102]. Another publication suggests a µ*g*-induced protein kinase C (PKC), known as a T-cell activation regulator. Compared to the 1*g* control cells, the production of interleukin-1 by U937 cells was significantly reduced in µ*g*, suggesting that the observed changes in PKC distribution may have functional implications [103].

An investigation was conducted using microarrays to study the effect of s-µ*g* on the expression of microRNA (miRNA) and related genes in human lymphoblastoid cells. The study involved culturing TK6 cells in a bioreactor for 72 h, either rotating or static. The results showed that µ*g* significantly altered global gene expression patterns and protein levels, which could regulate up to one-third of all human genes. There were also significant changes in miRNA expression, which correlated with gene expression and functional cell changes in several pathways, including immune/inflammatory response, NFκB pathway, p53 pathway, hypoxanthine-phosphoribosyl transferase gene, apoptosis, and survival pathway. Additionally, next-generation sequencing investigations of the same cell line after 48 h of simulation with rotating cell culture revealed DNA methylation, resulting in alterations in DNA-dependent transcription and carbohydrate metabolic processes [104,105,106,107,108,109,110].

The MOLT-4 lymphoblast leukemia cell line exhibited an altered cell shape, decreased viability, and an abnormal cell cycle profile under µ*g*. The genome-wide expression profiling revealed a significant post-transcriptional gene silencing machinery dysregulation and several microRNA host genes, including *MIR22HG*, *MIR17HG*, and *MIR21HG*. These genes are putative tumor suppressors and proto-oncogenes. Moreover, the genome-wide expression profile revealed that the tumor-suppressor gene *MIR22HG* was highly elevated, with a 4.4 log-fold increase under µ*g*. Real-time PCR confirmed the dysregulation in the host gene, showing a 4.18 log-fold increase of miR-22. The microarray data also revealed dysregulations of miR-22’s primary targets, *SP1*, *CDK6*, and *CCNA2*. The DNA microarray investigation of MOLT-4 cells revealed the differentially upregulated expression of 349 and downregulated expression of 444 genes (more than twofold) during µ*g* [111].

Numerous studies have utilized U937 cells to investigate the impact of µ*g* on their structure, adhesion, migration, proliferation, signal transduction, cytokine production, and immune response. These studies have also helped to identify the gene expression differences related to leukemic myelomonocytic cells [112,113,114,115,116].

Other researchers used an RWV bioreactor to simulate µ*g.* They observed a decrease in the expression of actin protein in U937 cells. Additionally, they detected a disorganization of the cytoskeleton and a decrease in cell proliferation. The study also revealed changes in cell shape and disruption of cytoskeletal structural proteins, namely vinculin and β-tubulin [114,116].

Compared to 1*g* controls stimulated with phorbol myristyl acetate, 12-*O*-tetradecanoylphorbol-13-acetate-stimulated U937 cells showed a reduced activation of c-jun and less tyrosine-phosphorylation. However, under s-µ*g* conditions, the U937 cells responded with an overall increase in tyrosine-phosphorylation and activation of c-jun. Additionally, it was observed that the p53 protein underwent a rapid phosphorylation in µ*g* [113].

To investigate the impact of µ*g* on growth retardation and reduced mitogenic activation, U937 cells were grown in the RWV. The study showed that cells grown in the RWV exhibited a slower growth rate than cells grown under 1*g* control conditions, which is consistent with the results of cells grown in space. Moreover, a notable decrease in proteasome activity and the protein array revealed changes in the cytokine secretion profile, particularly for inflammatory chemokines. The study also observed an upregulation of hsp70, which partially prevented apoptosis, and a down-modulation of cdc25B, which is linked to the slower growth rate of cells in µ*g* [116].

In µ*g*, hypoxia-inducible factor 1 (HIF-1) is differently regulated. Additionally, in human myelomonocytic cells, phosphoinositide-dependent kinase-1 (PDK1) is sensitive to gravitational changes. Meanwhile, MEK phosphorylation increases 1.3 times in U937cells [113,115]. Intercellular adhesion molecule 1 (ICAM-1) expression has increased in the U937 cells exposed to µg conditions, using 2D clinostats [112].

Using a microarray, the transcriptomic study found 1709 and 4667 differently regulated transcripts in U937 cells subjected to 22 s of µ*g* during parabolic flight or 300 s of µg during a suborbital rocket flight [117]. During that parabolic flight, transcripts associated with DNA replication and microtubule-based processes were affected. On the other hand, the suborbital flight caused changes in intracellular transport, mRNA processing, RNA and enzyme binding, post-translational regulation, cell cycle regulation, and cell division. Another study demonstrated that the ERK/MAPK pathway remained unaltered after 5 min of clinostat rotation in monocytic U937 cells. Moreover, there was only a negligible impact on NFκB activation [113].

Further studies have shown that s-µ*g* has the potential to exhibit an anti-cancer effect. When human HL (Hodgkin’s lymphoma) cells were exposed to time-averaged s-µ*g* for 2 d, they exhibited reduced intracellular ATP levels, mitochondrial mass, ATPase, ATP synthase, and increased ROS generation. Additionally, there was an increase in the expression of the NADPH oxidase family genes (*gp91*-, *p22*-, *p47*-, and *p67-phox*). In addition, the expression of these genes was upregulated (*ULK1*, *ATF4*, *Beclin-1*, and microtubule-associated protein one light chain 3 (LC3), whereas the expression of ATPase (*ATP1A1*) and ATP synthase (*ATP5A1*) was downregulated. The regulation of the AMPK/Akt/mTOR and MAPK pathways led to the induction of autophagy in HL cells exposed to s-µ*g*. However, this autophagy was blocked by the ROS scavenger acetylcysteine (NAC). It is known that autophagy plays a significant role in mediating the pathogenic response and the cell’s reaction to oxidative stress brought on by reactive nitrogen species and ROS. The creation of double-membrane-bound organelles known as autophagosomes, a byproduct of autophagy, is initiated by the phosphoinositide 3-kinase (PI3K) type III-Atg6/Beclin-1 cascade. The traditional PI3K/Akt/rapamycin-binding mammalian target pathway also involves autophagy [118]. Briefly, increased ROS production and NADPH oxidase family gene expression, mitochondrial mass and ATPase, ATP synthase, and intracellular ATP levels decrease were reported in HL cells exposed to µ*g*.

As previously noted, µ*g*-environments can profoundly impact human physiology at the cellular, organic, and systemic levels. Researchers conducted a study on Jurkat lymphocytes to explore the cellular-level effects of s-µ*g*. Jurkat cells serving as biosensors for the body’s responses were grown on an RPM and at 1*g*. Researchers employed cytofluorimetric and staining protocols to analyze the cells’ morphology, cell cycle, and proliferation. Additionally, fluorescent probes were used to measure O_2_^−^, intracellular Ca^2+^, ROS, mitochondrial membrane potential, aconitase, mitochondrial activity, glucose, and lactate levels. After the initial days of exposure, the cells exhibited a more uniform, spherical shape and a faster proliferation rate. They reduced intracellular ROS and Ca^2+^ levels due to metabolic and detoxifying activity. Throughout the late exposure period, the cells became acclimated to the changing ambient conditions [119].

After a 12 h RWV exposure, primary human blood NK cells were compared to static and vertical axis rotational controls. The study showed that s-µ*g* (horizontal axis rotation) decreased subsequent NK-cell cytotoxic function in 1*g* against allogeneic target cell lines of leukemia (K562 cell line), multiple myeloma (U266 cell line), B-lymphoma (721.221), and HLA-E transfected lymphoma (221. AEH) origin. However, surface expression of a wide range of NK-cell activating and inhibitory receptors remained largely unaffected, and intracellular perforin expression was decreased. Further analysis using CyTOF revealed that s-µ*g* lowered NK cell degranulation and reduced pro-inflammatory cytokine expression in response to the K562 co-culture at 1*g* [120].

As shown by DNA array analysis, previous reports have found that µ*g* can impact signal transduction in lymphocytes by modifying protein kinase C, Tor/CD3, NFκB, and MAPK signaling [103,121,122]. Rel/NFκB signaling was inhibited in µ*g*. Additionally, 47 genes, including the *CREB* and *SRF* genes, revealed a significantly downregulated expression after 1.5 h of human T cell activation in the spaceflight experiment [123]. Notably, further research has indicated that the expression of Fas in Jurkat cells increased during spaceflights on the STS-80 and STS-95 shuttle missions, suggesting an augmentation of Fas-FasL mediated apoptosis [124]. The decrease in PKC translocation observed in the U937 cell line has significant implications for regulating apoptosis and cell cycle arrest. As a critical regulator of these processes, PKC translocation is crucial in maintaining cellular homeostasis. Therefore, the observed reduction in PKC translocation highlights a potential mechanism for the dysregulation of these processes in the U937 cell line [122]. The use of a 3D clinostat has shown that µ*g* significantly inhibits the growth of two different human Hodgkin’s lymphoma cell lines (L-540 and HDLM-2) when compared to the proliferation of natural human dermal fibroblast cells (HDF), which remain unaffected [125].

In addition, Jurkat T cells, a type of human white blood cell used to study acute T cell leukemia, exhibit elevated levels of cytochrome c, *µ*-calpain expression, and activity of apoptosis-inducing enzymes when exposed to s-µ*g*. These changes are linked to an imbalance of interleukin (IL-) 2 and interferon (INF-) *γ* cytokines. The interaction between these cytokines is responsible for the observed imbalance, while *µ*-calpain activity playing a significant role in the process. Inhibiting *µ*-calpain activity can help to prevent apoptosis, modify the IL-2/INF-*γ* ratio, and prevent the activation of *µ*-calpain induced by s- µ*g* [126]. Using proteomics techniques on Jurkat cells exposed to µ*g*, a decrease in IL-2 production and cell proliferation was found. This was due to the cells’ failure to enter the cell cycle. The levels of nucleic acid production were reduced because of the weakened function of the transmembrane C-type lectin protein encoded by the *CD69* gene. In addition, there was a reduction in the mitochondrial membrane potential, which resulted in the downregulation of ATP-dependent DNA helicase and other cellular signal transduction [108]. As mentioned in one study, the µ*g*-dependent activation of 5-lipoxygenase by the lipoxygenase pathway in K562 cells might be essential in initiating apoptosis [127,128]. The expression of the prostaglandin H synthase enzyme in the same leukemic cell line K562 was significantly increased by hypergravity [129].

Another publication reported about Jurkat T lymphocytes from human acute leukemia which were sent into space on the STS-95 to observe the impact of a space travel on the expression of cytoskeletal genes. mRNAs were evaluated using a cDNA microarray after 24 h (4324 genes) and 48 h (>20,000 genes) during spaceflight and in-ground controls. In comparison to the control cells on Earth, transcripts of gelsolin precursors were reduced in space cells, along with calponin, dynactin, tropomodulin, keratin 8, two myosins, ankyrin EST, actin-like proteins, and cytoskeletal linkers. In contrast, transcripts of 11 cytoskeleton-related genes, including plectin and centriole-associated protein (C-NAP1), were increased. A unique discovery was made in that plectin and C-NAP1 messages may play a role in repairing vibrational damage, as both proteins were upregulated in spaceflight and vibrationally controlled cells [130]. Another research team investigated Jurkat cells exposed to µ*g.* The microarray revealed a suppression of 91 genes’ activation in contrast to normal gravity controls [121]. Shao and colleagues conducted a study in 2021 using K562 to investigate the effect of s-µ*g* on the cytotoxicity of natural killer cell lines (NK). They used bisulfite modification, DNA sequencing, and real-time PCR to analyze the expression of stimulatory and inhibitory receptors. Their findings suggest that the inhibition of the cytotoxicity of NK cells is due to the downregulation of the receptor NKG2D and its adaptor protein DAP10 rather than DNA methylation [131].

To examine how Jurkat cells react to vibrations in space, the genes *HSPA1A, HSPA1B* and *HSPB1* were analyzed through reverse transcription polymerase chain reaction. The results indicated that low gravity did not cause a rise in *HSPA1aA and HSPA1B* mRNA but did result in increased gene encoding of *HSPB1*. Conversely, the mRNA expressions of both heat shock proteins increased in the ground control samples [132]. The protein FAS/APO-1, also known as the apoptosis antigen 1, is responsible for the process of apoptosis. Jurkat cells exposed to a space shuttle flight have shown an abnormal behavior, such as increased glucose metabolism and microtubule organizing centers. Moreover, the expression of this protein has increased over time due to µ*g* conditions [124,133].

µ*g* affects the proliferation of human leukemic (Jurkat) cells and the expression of their survival-controlling receptors, such as VEGFR-1, VEGFR-2, and VEGFR-3 [134].

The structure of the filamentous protein vimentin was examined in Jurkat cells during a sounding rocket flight. Both the formation of big bundles mentioned to changes in the design of vimentin and whether actin’s structure and its colocalization with Con A receptors on the inner side of the cell membrane following mitogen binding are unaltered, suggesting that the modifications were limited to the membrane and most likely did not affect the cytoskeleton [135].

Researchers used RWVs to study their effect on nonadherent promyelocytic (HL-60) cells from females with acute promyelocytic leukemia. The study found that under µ*g*, these cells exhibited reduced respiratory impulses (ROIs) and an altered time-dependent production of ROIs. Moreover, only 9.8% of the s-µ*g* cells expressed the CD11b antigen compared to 12.1% of the HL-60 cells under normal 1*g* conditions. This suggests that HL-60 cells exposed to s-µ*g* may undergo less differentiation [135].

Furthermore, Wang et al. [136] used a RCCS bioreactor to determine its effects on polymorphonuclear neutrophils (PMN)-like HL-60 cells. The observed alterations in cellular morphology, heightened nitric oxide synthesis, augmented secretion of monocyte chemotactic protein 1 (MCP-1), interleukin-6 (IL-6), and interleukin-8 (IL-8), along with a varied expression of cellular adhesion molecules. These changes were explicitly noted in cells cultured in RCCS, which increased inflammatory immune responses and host defense [136].

Another analysis examined the same cell line using a DNA fragmentation assay and semi-quantitative rt-PCR. The findings indicated an increase in DNA damage and the presence of DNA damage-sensing proteins such as ATM, ATR, Chk1, Chk2, and γH2A. Furthermore, there was a shift in the overall DNA repair capacity, as differentially expressed DNA repair genes AP1, XRCC1, and APEX1 (which regulate base excision repair) were upregulated. In contrast, XPC MLH1 and PMS2 (which regulate mismatch repair) were downregulated when subjected to s-µ*g*. Additionally, the investigation discovered heightened levels of cleaved-poly-(ADP-ribose) polymerase and cleaved caspase-3, an increase in the Bax/Bcl-2 ratio, and dissipation of mitochondrial membrane potential, leading to apoptosis [137].

When subjected to s-µ*g* using a rotary culture, an experiment on the UT-7/EPO leukemia cell line showed a noticeable decrease in surface localization, protein content, and mRNA expression of the erythropoietin receptor (EPOR). Additionally, the experiment revealed the activation of caspases-3 and the downregulation of Bcl-xL was due to s-µ*g* [138]. When the human EPOR gene was transferred into UT-7/EPO cells, the expression of EPOR on the surface of these cells increased by approximately 61% (*p* < 0.05), as confirmed by antibiotic selection. Further cytometry assays and morphological observations revealed that µ*g*-induced apoptosis significantly decreased in these UT-7/EPO-EP cells [138].

Taken together, these publications increased the current knowledge concerning the effect of µ*g* on hematological cells and blood cancer cells, as well as our understanding of human lymphoma cell mechanisms. These results could lead the way to find new treatment strategies for cancer patients.

### 3.3. Sarcomas

In contrast to carcinomas, sarcomas are relatively rare malignant tumors of mesenchymal origin. Overall, they account for less than 1% of new cases of solid malignant cancers. While the absolute number of cases increases with age, relative shares for sarcomas are highest among pediatric cancers (7–20%) and decrease rapidly with time. Sarcomas are a very heterogenous malignancy, with about 100 distinct histologic subtypes. By far the most common types are soft tissue sarcomas (STS) in muscles, joints, fat, nerves, deep skin tissues, and blood vessels with about 87% of all sarcomas. The remaining 13% comprise bone tumors such as osteosarcomas and chondrosarcomas. In particular, STS have a relatively bad prognosis. This is mainly due to the fact that early stage STS has unclear symptoms so that most are diagnosed at an advanced stage, often with metastasis [139,140,141,142].

Kunisada et al. exposed the human osteosarcoma-derived cell line Hu09 to s-µ*g* on a horizontal clinostat for 7 d. They detected no morphological changes, and the total amount of produced protein was unaltered. However, both alkaline phosphatase activity as well as osteocalcin production were significantly reduced under s-µg conditions. An addition of 0.1 µM 1,25-dihydroxy-vtamin D3 to the culture medium induced an increase in alkaline phosphatase (ALP) activity in both 1*g* control and s-µ*g* samples. While Hu09 cells on the clinostat showed a stronger reaction to this stimulus, the overall ALP activity in µ*g*-exposed cells still remained significantly lower than in 1*g* cells. Furthermore, 0.1 µM 1,25-dihydroxy-vtamin D3 (1,25(OH)_2_D_3_)-induced secretion of bone γ-carboxyglutamic acid-containing protein (BGP) was also significantly reduced in clinorotated cells. The authors concluded that s-µ*g* directly inhibits Hu09 differentiation and functions [143].

Another experiment in s-µ*g* employed the Ewing’s Sarcoma cells line A673 cultured on the RPM for 24 h. The authors observed spheroid formation under s-µ*g* in addition to the adherent cells and found that *CXCR4* and *CD44* gene expressions were significantly enhanced in spheroids only, while *EWS/FLI1* and *CAV1* were significantly upregulated and *DKK2* and *VEGFA* significantly downregulated in both adherent and spheroid samples compared to 1*g* controls. Protein concentration of EWS/FLI1 was only elevated in RPM adherent cells. On the other hand, inhibition of the chemokine receptor *CXCR4* did not affect spheroid formation [144].

Lastly, MG-63 cells were used in slow-turning lateral vessels (STLVs), a rotating cell culture system that leaves cells in constant free fall, thus simulating µ*g*. It was found, that *COL1A1* gene expression, as well as osteocalcin, ALP, and vitamin receptor (VDR) protein levels, were reduced in s-µ*g* samples, and that these cells were less responsive to 1,25(OH)_2_D_3_. Moreover, growth in STLVs activated the VDR activity-inhibiting stress-activated protein kinase pathway, which could be compensated to some extent by supplementation with EB1089, a 1,25(OH)_2_D_3_ analog. Overall, the authors concluded that s-µ*g* reduced both differentiation and VDR activity in MG-63 cells [145].

In addition to these studies, experiments with sarcoma cells in r-µ*g* have been conducted. Human osteosarcoma MG-63 cells were flown for 9 d in space on board the FOTON-10 satellite. µ*g* samples were either untreated or received hormone supplementation (0.1 µM 1,25(OH)_2_D_3_; 10 ng/mL transforming growth factor b2) and were compared to 1*g* ground controls as well as in-flight 1*g* controls. The authors found that the increase in ALP activity following hormone treatment was significantly less pronounced in µ*g* samples compared to 1*g* controls (1.8-fold increase vs. 3.8-fold increase, *p* < 0.01), while no difference was observed for collagen type I production. Interestingly, *COL1A1*, together with *BGLAP* and *ALPL* gene expressions in space-flown samples, were significantly reduced compared to 1*g* cells. Moreover, the *ALPL* gene expression increase after hormone treatment was significantly smaller in µ*g* cells vs. 1*g* controls (5-fold vs. 10-fold, *p* < 0.02). Overall, the response to hormones and growth factors as well as differentiation was reduced in µ*g* [146,147].

Guignandon et al. exposed MG-63 cells to 69 h of µ*g* on the FOTON M3 satellite. The RhoGTPases RhoA, Rac1, and Cdc42 were silenced by specific siRNAs (*SiRhoA, SiRac1*, and *SiCdc42*, respectively), with scrambled SiRNA (*SiScr*) as control. After the flight, SiScr cells showed reduced focal adhesions and actin stress fibers. The same pattern was found for µ*g*-exposed SiCdc42 and SiRhoA cells, but not for SiRac1 cells, which exhibited no elevated cell detachment, a spread phenotype, and well-developed actin stress fibers. In space, fibrinogen was significantly reduced (up to −50%) in all cell types except for SiRac1 cells (−20%, *p* > 0.05). Gene expression analyses for the three VEGF-A isoforms VEGF-A_121_, VEGF-A_189_, and VEGF-A_165_ showed that under 1*g* control cells and the Rho GTPase-silenced cells expressed all three isoforms similarly. In µ*g*, however, mRNAs for VEGF-A_121_ and VEGF-A_165_ were significantly increased and mRNA for VEGF-A_189_ was significantly decreased in both SiScr and SiRhoA cells. SiCdc42 cells only showed VEGF-A_165_ upregulation, and no differences were observed for SiRac1 cells. Lastly, levels of matrix-bound VEGF were significantly reduced in SiScr, SiRhoA, and SiCdc42 cells, but did not change in SiRac1 cells under µ*g* conditions. Overall, the authors concluded that the VEGF/Rho GTPase axis plays an important role in mechanosensing and identified the Rac1 pathway as a promising target for counteraction µ*g* effects [148].

### 3.4. Thyroid Cancer

In contrast to prostate cancer, thyroid cancer occurs more frequently in younger people. According to the American Cancer Society, the average age at first diagnosis is 51. Thyroid cancer is three times more common in women than in men [149].

The five-year survival rate depends on the occurrence of distant metastases, but also in particular on the tumor type. Papillary and follicular thyroid carcinomas have a relatively high five-year survival rate even in the presence of metastases (~70%), while this is greatly reduced in anaplastic thyroid carcinomas.

In µ*g*-research, the three thyroid carcinoma cell lines, ML-1, RO82-W-1, and FTC-133, have proven to be frequent subjects of analysis.

In 2002, Grimm and coworkers [150] exposed the ML-1 thyroid carcinoma cell line to s-µ*g* using a 3D clinostat. Western blot analyses revealed an increase in extracellular matrix proteins (collagen I, III, fibronectin, laminin, and chondroitin sulfate) in µ*g*-MCS. Furthermore, immunofluorescence analysis showed an increase in vimentin-positive cells, and an increase in thyroid-stimulating hormone (TSH) receptor. After 24 h of clinorotation, flow cytometry revealed a decrease in thyroglobulin content and an elevation of the apoptosis markers Fas/Apo-1 and p53. This increase was interpreted by the authors as an indication of programmed cell death initiation in conjunction with the detection of an 85-kDa apoptosis-related cleavage fragment of PARP, reduced fT3, fT4 secretion, and clear morphological signs.

In 2011, Ulbrich et al. [151] reported results of the exposure of ML-1 thyroid carcinoma cells to the altering gravity conditions of a parabolic flight (PF). They performed gene expression array and qPCR analyses to receive transcriptional insights into the role of the interaction between the cell and its microenvironment in cancer progression. They found 148 significantly regulated genes in the array experiment. Already after the first parabola (22 s µ*g*), they observed an upregulation of cytoskeletal genes (*ACTB* and *KRT80*) in conjunction with a downregulation of *COL4A5*, which codes for the α-5 subunits of collagen type IV. Ground experiments under 1.8*g* hypergravity and simulated flight vibrations did not change the expression of those three genes. In summary, the authors found transcriptional confirmation of the severe effects of µ*g* on the cytoskeleton. 

Svejgaard and coworkers [152] exposed ML-1 cells and RO82-W-1 cells, derived from the metastases of a follicular carcinoma, to µ*g* using an RPM and a 2D clinostat, respectively (3- and 7-d s-µ*g*-exposure). Unlike ML-1, RO82-W-1 cells exhibited no change in β-actin in s-µ*g*. RPM and the 2D clinostat-exposure showed similar effects on ML-1 and RO82-W-1 cells both in terms of cytokine release and cytoskeletal protein expression. In ML-1 cells, the two s-µ*g* applications differed only for 2 out of 14 released cytokines.

In contrast, Riwaldt et al. [153] observed, after 24 h of s-µ*g* exposure (RPM) of RO82-W-1 cells, alterations in the F-actin cytoskeleton. In RO82-W-1 MCS, they observed an *VEGFA*, *VEGFD*, *MSN*, and *MMP3* upregulation. Pathway analyses revealed the associated proteins to promote 3D growth (angiogenesis), while genes coding for structural proteins are downregulated.

A first free flow isoelectric focusing (FF-IEF)-based proteome analysis of the cell line FTC-133 under s-µ*g* condition (RPM, 72 h) was performed by Pietsch and coworkers in 2010 [154]. FTC-133, which was originally obtained from a lymph node metastasis of a follicular thyroid carcinoma, showed a downregulation of the protein expression of alpha-enolase, phosphoglycerate kinase 1, annexin 1, and 2 under RPM exposure in the subsequent quantitative analysis of Western blot analysis with a simultaneous upregulation of the protein content of glutathione S-transferase.

Cytometry revealed a significant twofold increase of apoptotic and necrotic cells in adherent cells already after a 24 h RPM-exposition of FTC133 [155]. Using Western blotting, it was found that the NFκB component RelA (p65), which is involved in the body’s inflammatory response, is upregulated in MCS. Additionally, the NFκB interactors CTGF, TLN1, IL-6, IL-8, CD44, and SPP1 are significant regulated by s-µ*g* on the gene level (gene array and qPCR). The authors suggest an initiation of an early phase of apoptosis during transit from 2D to 3D cell growth. The gene array analysis revealed an upregulation of 13 ribosomal protein coding genes in adherent cells after 24 h of RPM exposition, indicating a general increased expression and translational activity. At the same time, genes with high expression in MCS are widely unrelated and consist of several transcription factors, the cell adhesion-related protocatherins β5 (PCDHB5) and 7 (PCDH7), the cell surface-bound chemokine CX3CL1 and RAPGEF3 which has a role in the integrin activity control. The glycosphingolipid biosynthesis was enriched in µ*g*-induced downregulated genes.

On the first of November 2011, the Shenzhou-8 spacecraft was launched from the Jiuquan Satellite Launch Center in Northern China. On board were FTC-133 cells from which spheroids were grown during the mission in r-µ*g* and in an automated culture system (ACS) [156]. Pietsch et al. studied the regulation of *EGF* and *CTGF* gene expression in r-µ*g* (ACS) and s-µ*g* (RPM) under the same unusual temperature conditions (23 °C) that were specified by the space mission. They observed an increased EGF and CTGF expression under r-µ*g* and s-µ*g*. From the similar MCS and AD expression pattern, the authors conclude that there is increased evidence for the role of the two growth factors in the transition of thyroid cancer cells from 2D to 3D growth. The study by Ma and coworkers [42] is dedicated to the integration of effects of short-term µ*g* exposure (PF) with experiments of longer-term µ*g* exposure (Shenzhou-8 space mission) based on the thyroid tumor cell line FTC-133. They found that key genes involved in tumor cell proliferation and metastasis formation (e.g., *IL6*, *CXCL8*, *IL15*, *OPN*, *VEGFA*, *VEGFD*, *FGF17*, *MMP2*, *MMP3*, *TIMP1*, *PRKAA*, and *PRKACA*) in the Shenzhou 8 space mission responded similarly to µg in subsequent ten-d RPM experiments in an antiproliferative manner with often opposite effects in PF. The authors emphasized the benefit of accompanying s-µ*g* experiments. Accordingly, Warnke et al. [157] subsequently investigated the frequently used s-µ*g*-platforms 2D clinostat and RPM in their effect on spheroid development and accompanying expression changes in 16 genes (qPCR). The expression depletion of *CAV1* and *CTGF* in MCS compared to adherent cells was observed on both machines. This led the authors to the conclusion that both platforms mimic spheroid formation physiologically sufficiently similar to r-µ*g* conditions.

SpaceX CRS-3, a Commercial Resupply Service mission to the International Space Station (ISS) launched on 18 April 2014, and carried FTC-133 cells in a similar ACS as used earlier on the Shenzhou-8 mission. The cells returned to Earth with a reentry vehicle landed on 20 May 2014, [158]. Surprisingly, no macroscopic differences between in-flight and ground-control cultures could be observed, the in-flight FTC-133 was found to be monolayer cultures without MCS development. The authors hypothesize the unexpected prolonged pre-incubation time as a reason. Mass spectrometry led to 47 proteins unique in ground control cells and 13 proteins unique in the flown cells, respectively. Riwaldt and coworkers [159] found evidence for a key role of caveolin-1 protein in the inhibition of spheroid formation when confluent monolayers are exposed to µ*g*. They analyzed the protein content of in-flight FTC-133 monolayer cultures using mass spectrometry and the supernatant by Multi-Analyte Profiling (MAP) technology. A protein interaction analysis on soluble and cell-associated proteins revealed plasminogen as central interactor, where plasminogen itself facilitates cancer cell migration as well as spheroid formation. Exosomal secretion of FTC-133 cells was analyzed by Wise et al. [160] to determine number and size distribution of secreted vesicles and their population regarding the tetraspanin surface expression to finally enlighten changes in cellular cross-talk under µ*g*. The authors found µ*g* induced differences in the number of secreted exosomes, in the distribution of subpopulations and alteration of their population regarding the tetraspanin surface expression. By Wise et al. [161] additional changes in the exosomal miRNA composition have been observed. Of 754 miRNA targets analyzed in an array scan 119 miRNAs have been found to be differentially expressed due to the µ*g*-effect. Of these miRNAs 19 had a higher relative quantification (RQ) under r-µ*g* condition and 100 had a decreased RQ value.

The commercial resupply service mission SpaceX CRS-13 to the ISS launched on 15 December 2017. On board were FTC-133 samples in a CellBox-2 incubator which, in contrast to the SpaceX CRS-3 mission, ensured a temperature of 28 °C for the cells [162]. After 5 d or 10 d of r-µ*g*, the cells where fixed and after return to Earth the RNA levels of 19 key genes where measured and the secretion of 25 proteins were quantified (MAP or ELISA). Of 17 secreted factors with sufficient concentration, only angiopoetin-2 was altered. The authors found suppression of *VCL*, *PXN*, *ITGB1*, *RELA*, *ERK1*, and *ERK2* gene expression under r-µ*g* conditions and a downregulation of *MIK67* and *SRC* in MCS and identified the ERK/RelA pathway as a major µ*g* regulator.

Differences between Nthy-ori 3–1 normal thyroid cells and FTC-133 were observed in the expression of the genes *NGAL*, *VEGFA*, *OPN*, *IL6*, and *IL17* and the secretion of VEGF-A, IL-17, and IL-6 in a 14-d RPM-experiment [163]. Kopp and coworkers found a different growth behavior for both cell lines. The most noticeable difference was the smaller MCS size in normal Nthy-ori 3-1 cells. The authors assume that growth and angiogenic factors determine the differences in the spheroid formation behavior of malignant and healthy thyroid cells.

The DLR TEXUS-53 sounding rocket mission took place in January 2016. The sounding rocket enabled Kopp and co-workers to expose FTC-133 cells to a 6 min µ*g*-phase during the flight [164]. They found the cytoskeletal genes *ACTB*, *TUBB1*, and *VIM* and genes coding the proteins of the ERM (ezrin-radixin-moesin) group downregulated by r-μ*g*. The ERM proteins are known to crosslink actin with the cellular plasma membrane. A moderate *LAMA1* expression reduction was observed under µ*g*. Its gene product located in the extracellular matrix and is involved in cell adhesion. Additionally, they observed a downregulation of the apoptosis-related genes *BAX* and *BCL2* and of *EGF* encoding the epidermal growth factor. To determine the influence of the one-minute 18*g* phase of the sounding rocket flight, Kopp et al. [165] centrifuged FTC-133 cells and found moderate but significant gene expression changes in *COL1A1*, *VCL*, *CFL1*, *PTK2*, *IL6*, *CXCL8*, and *MMP1*4. They concluded that µ*g* is the stronger gene expression regulator compared to hypergravity.

Dexamethasone (9-fluoro-16α-methylprednisolone) is an artificial long-acting anti-inflammatory glucocorticoid. Melnik and coworkers treated RPM-exposed FTC-133 cells with dexamethasone to investigate the observed dose-dependent suppression of spheroid formation at the transcriptional level [166]. They observed a heterogeneous reaction of NFκB components to dexamethasone treatment which could not explain the inhibition of μ*g*-triggered spheroid formation. However, the authors found evidence for complex interactions between proteins of the Wnt/β-catenin and TGF-β metabolic pathways. Melnik et al. [167] expanded their experimental portfolio with the benign cell line Nthy-ori 3-1 and the cell line WRO, established from the metastases of a follicular carcinoma, and with additional genes and proteins of the field’s extracellular matrix, cell junctions, adhesion, and signaling. They conclude that cell detachment selectively suppressed by DEX under s-µ*g* in metastatic cancer cells supported by massive tight junction formation, whereas in non-metastasis cells the upregulation of anti-adhesive mucin-1 plays an important role.

Taken together, the focus of µ*g*-based omics analyses in thyroid carcinomas is diverse and ranges from transcriptional to proteomic and secretomic to exosomic experiments. Even regulatory approaches can be found in miRNA determinations.

### 3.5. Prostate Cancer

Prostate cancer (PC) is the second leading cause of cancer-based death in men after lung cancer. The American Cancer Society [168] forecasts 288,300 new PC cases and 34,700 PC deaths for the USA in 2023. The 5-year survival rate drops dramatically from almost 100% to 32% when distant metastases occur. These statistics imply a high relevance of early detection of this disease, which can be ensured by suitable biomarkers, i.e., early onset biomarkers. The modernization of tumor diagnostics is therefore important due to the poor prognosis of metastasizing prostate carcinomas. In particular, these innovations can be achieved through systematic research into accompanying omics changes.

The early studies on the influence of µ*g* on PC focus on single to a few proteins that were mostly analyzed with immunocytochemical methods. Due to this limitation, they are not omics studies in the strict sense, but have decisively shaped the understanding of the biochemistry of 3D-based prostate carcinoma pathology and therefore deserve mention in this context.

Already in an early study from 1996 [169], the effects of s-µ*g* on the immunocytochemistry of DU-145 PC cells were investigated by Clejan and coworkers. DU-145 cells derived from a central nervous system metastasis, of a primary prostate adenocarcinoma. In this study, they analyzed cells cultivated on the high aspect rotating-wall vessel (HARV) in suspension on microcarrier beads (Cytodex-3). The authors observed extensive three-dimensional growth of the cells between the beads and on the surface of bead aggregates. This was particularly evident after 11 d with a drastic increase in cytokeratin 18, actin, and vimentin concentrations.

One year later, Zhau and colleagues [170] investigated the effect of s-µ*g* on the androgen-sensitive human prostate adenocarcinoma cells line LNCaP using the low-turning lateral vessel (STLV) version of a rotating wall vessel. LNCaP cells were derived from a supraclavicular lymph node metastasis. With the addition of dihydrotesterone (DHT), they observed a steady increase in cell numbers under 2D conditions and an associated increase in the production of prostate-specific antigen (PSA). These effects were more moderate under s-µ*g*. The sustained increase in PSA production could only be achieved in co-culture with human prostate fibroblasts. The authors noted that DHT promotes growth and differentiation of LNCaP that mimicked the course of androgen in vivo.

In the same year, Ingram et al. [171] published the three-dimensional growth pattern of various tumor cell-lines, including those of the prostate tumor cell-lines LNCaP, DU-145, and PC-3. PC-3 cells were originally derived from a prostate cancer bone metastasis. Compared to monolayer cultures, the authors generally observed a profound change in cell shape and configuration in the three-dimensional structures from the HARV bioreactor. Using immunohistochemistry, they found an increased expression of E cadherin and CD44 in the LNCaP 21d spheroids as compared to 1*g* controls.

In 1999, Margolis and colleagues [172] exposed mainly healthy prostate tissue to an RWV. Using immunohistochemistry, the levels of cytokeratin, vimentin, and TGF-P2 receptors and ligand were maintained after 28 d of s-µ*g*. While prostatic acid phosphatase (PAP) levels were consistently but slightly decreased, the authors found PSA to be downregulated. Under s-µ*g*, the prostatic carcinoma TSU-PR1 cell line, derived from a lymph node metastatic prostate adenocarcinoma and characterized by a low PAP level, was able to invade benign prostate tissue. PAP levels showed no significant changes in PAP levels in a seven-d dry immersion experiment in healthy volunteers [173]. The dry immersion bath is used to simulate µ*g*-aspects in test subjects.

Clejan and colleagues [174] investigated the differences between a 3D growth model (HARV) and 2D dual layer growth in DU145 PC cells. They summarized the general characteristics of the HARV model as less aggressive and proliferative, slower growing and more highly differentiated. They considered the cause to be an interaction between ceramides and the PIK3 pathway and an imbalance in phospholipase metabolism and an increase in cyclic adenosine monophosphate (cAMP).

The first study on PC cells in r-µ*g* took place in 2003 as part of the Columbia Space Shuttle mission. Due to the tragic end of the mission, which resulted in the death of the seven astronauts, the PC experiment carried on board could not be finally evaluated. It is known that the experiment carried out by astronauts Laurel Clark and Kalpana Chawla revealed three-dimensional structures of huge size [175].

The following publications focus on systematic transcriptional characterization of the influence of µ*g* on PC cells using qPCR) [176,177,178] and next generation sequencing (NGS)-based RNA sequencing (RNAseq) [176].

Hybel et al. [178] investigated the influence of a 3- and 5-d RPM-exposure of PC-3 cells on spheroid development. The authors used qPCR to investigate genes associated with the cytoskeleton, adhesion, growth, survival, angiogenesis, and carcinogenesis. The significant downregulation of gene expression of *VEGFA*, *SRC1*, *AKT*, *MTOR*, and *COL1A1* in PC-3 cells after 3 d on the RPM indicates an impairment of the PI3K/Akt/mTOR signaling pathway, cell migration (VEGF), and cell growth (SRC1, COL1A1). This, in combination with the significant decreases of secreted VEGF and NGAL, leads the authors to conclude a less aggressive phenotype as a result of the RPM-cultivation. In contrast, the study enlightens a significantly upregulated *ERK1/2* expression after 5 d on the RPM in both, AD and MCS which indicates a context of promoted tumor progression. In addition, both *FN1* and *VCL1* were upregulated in MCS after a 3- and 5-d RPM-exposure. The *FN1* regulation is attributed by the authors to its key role in the adaptation of podocytes to mechanical stress.

The study by Dietrichs et al. [177] had a similar approach, but here PC-3 cells were exposed to shorter RPM intervals (30 min, 2 h, 4 h, and 24 h). Controls, adherent cells, and MCS of PC-3 cells and the remaining cell culture supernatant were analyzed by qPCR and multiplex magnetic bead panel, respectively. After 24 h of RPM-exposure of the PC-3 cells, the science team observed a significant increase in 12 gene expressions in MCS including genes of the cytoskeleton (*ACTB*, *MSN*), genes coding cell surface components (*COL1A1*, *FN1*), three cytokines (*IL1A*, *IL6*, *CXCL8*) genes coding proteins with a function in migration (*LAMA3*, *TIMP1*, *FLT1*), the angiogenesis gene *HIF1A* and the gene encoding the epidermal growth factor receptor 1 (*EGFR1*).

The significant increases in the *IL6* and *CXCL8* gene expression of the RPM samples corresponds to the likewise significant increase in expression of the two cytokines after 22 s r-µ*g* in PC-3 cells exposed to PF maneuvers described by Schulz et al. [176]. Here, the PC-3 transcriptome (RNAseq) was determined after one parabola and after 31 parabolas and compared with each other and with ground controls. Via qPCR the expression of key genes of carcinogenesis was determined under r-µ*g*, under simulation of airplane vibration (vibraplex) and under simulation of the hypergravity phases of parabolic flight (Multi-Sample Incubation Centrifuge; MuSIC). Vibraplex and MuSIC each identified only one of the key genes as differentially expressed (*IL6*, *PIC3CB*). It can therefore be assumed that the simulated factors play a subordinate role. The analysis of the RNAseq data led to a list of 298 gravisensitive genes with a significant functional enrichment of cytokines and in particular chemokines. The *RELB* proto-oncogene NFκB subunit and the majority of c-Rel knock-out-dependent upregulated genes [179] were regulated in PF [176]. In contrast to the cytokine cluster HSPA1A and HSPA1B two 70 kDa heat shock protein coding genes are downregulated during the PF.

Taken together, the last two studies have identified differentially regulated cytokine genes as an early response to gravitational changes, whereas most of the genes or proteins mentioned in the 10 studies are known to play a role in carcinogenesis.

### 3.6. Breast Cancer

Breast cancer (BC) is the most common cancer and accounts for 12.5% of all new annual cancer cases worldwide [180]. In 2023, an estimated 297,790 new cases of invasive BC are expected to be diagnosed in the U.S. for women [180]. A recent meta-analysis published data from 2.4 million women with BC from 81 countries [181] showed that the proportion of cases with distant metastatic BC at diagnosis was high in sub-Saharan Africa and low in North America.

Metastatic BC is still an unsolved problem of this cancer during the last century and still represents a health burden with no effective healing strategy identified so far. Therefore, new ideas and innovations are mandatory to fight BC. For some time, BC cells have been studied under µ*g* conditions in real space and frequently on simulation devices which have been described in detail in Section 2.

A large number of publications have demonstrated that µ*g* has various effects on cancer and normal cells; by influencing adhesion, proliferation, migration, growth, and differentiation changes, among others, toward a less aggressive phenotype. In addition, µ*g*-research delivers a reliable in vitro 3D tumor model for preclinical cancer drug development and to study various processes of cancer progression. For this purpose, numerous molecular biological studies have been performed.

The search of publications provided 19 publications about research studying proteins, genes, soluble factors like cytokines in BC cells exposed to µ*g* conditions.

Early investigations focusing on different cancer cell lines exposed to a NASA rotary cell culture system demonstrated 3D growth [171]. The authors reported 3D multicellular spheroid formation in BT20 BC cells [171]. Human well differentiated MCF-7 BC cells flown in space in a Foton capsule were studied for 1.5 h, 22 h, and 48 h in orbit. Cells subjected to r-µ*g* revealed a prolongation of mitosis and an alteration of the cytoskeleton [182,183].

Coinu et al. [184] focused on vascular smooth muscle cells and transformed BC cells exposed to s-µ*g* by comparing cell proliferation, glucose transport, methionine uptake and protein synthesis. Modeled µ*g* profoundly affects cell growth and glucose or methionine metabolism. Both cell types express insulin receptors. The measured metabolic changes were strongly enhanced when the cells were simultaneously stimulated with insulin and subjected to µ*g* stress [184]. In 2006, the same authors showed that a 48 h lasting RPM-exposure changed the cytofluorimetric profile of MCF-7 cells and slows down fundamental metabolic activities (glucose uptake, methionine uptake/incorporation, and thymidine incorporation) in normal and transformed cells [185]. The RPM-exposure did not change the expression of most proteins that are related to the cell cycle and apoptosis, whereas it altered the expression of HSP-60, HSP-70, and 14-3-3 protein. These two studies are the only investigations of metabolic changes in BC cells exposed to µ*g*. The majority of the available literature focuses on gene expression changes or alterations of proteins in BC cells cultured under µ*g*.

Most publications demonstrate the well-known effects on morphology, cell functions, altered cytoskeletal, and adhesion structures. These changes are accompanied by altered biochemical pathways and gene expression alterations. µ*g*-exposed MDA-MB-231 BC cells revealed cytoskeletal alterations together with functional changes in biological processes (proliferation and apoptosis) and signaling pathways (ERK, AKT, and surviving) [186].

MCF-7 BC cells exposed to an RPM for five d revealed a downregulation in *ACTB*, *TUBB*, *EZR*, *RDX*, *FN1*, *VEGFA*, *FLK1, CASP9*, *CASP3*, and *PRKCA* mRNAs indicating the involvement of the cytoskeleton, extracellular matrix proteins, caspases, growth factors, and protein kinases in spheroid formation [95].

The data of a proteome analysis of RPM-exposed MCF-7 BC cells and static 1*g* controls was published in 2018 [187]. Most detected proteins showed similar label-free quantification (LFQ) scores in each of the respective subpopulations. There were also proteins in the AD and MCS group with LFQs deviated at least 2-fold compared with 1*g* control cells. For example, E-cadherin was reduced in MCS cells, but proteins of the E-cadherin auto-degradation pathway were enhanced and c-Src (proto-oncogene tyrosine-protein kinase c-Src) was also measured [187].

In order to study the mechanisms responsible for spheroid formation, the proteome analyses of FTC-133 human thyroid cancer cells and MCF-7 BC cells were re-analyzed by the use of semantic methods. Bauer et al. [188] focused on posttranslational modifications (PTMs) of proteins. Protein candidates showing a significant accumulation in spheroid cells as compared to 1*g* monolayer cells were selected. In total, the authors detected 72 different classes of PTMs comprising mainly phosphorylation, glycosylation, ubiquitination, and acetylation. In 35 of the 69 proteins, N6 residues of lysine are modifiable [188].

Another study [189] focused on extracellular vesicles (EV) derived from MDA-MB-231 BC cells in µ*g* using the gravity controller Gravite. Their data showed that compered to 1*g* control cells, the EV release rate decreased in µ*g* while the average EV size increased. EVs may be superior to cells in analyzing differentially expressed proteins. The proteomic analysis of both EVs and cells further revealed a significant correlation with GTPases and proliferation of MDA-MB-231 triple-negative BC cells in µ*g* [189].

**Figure 4:**
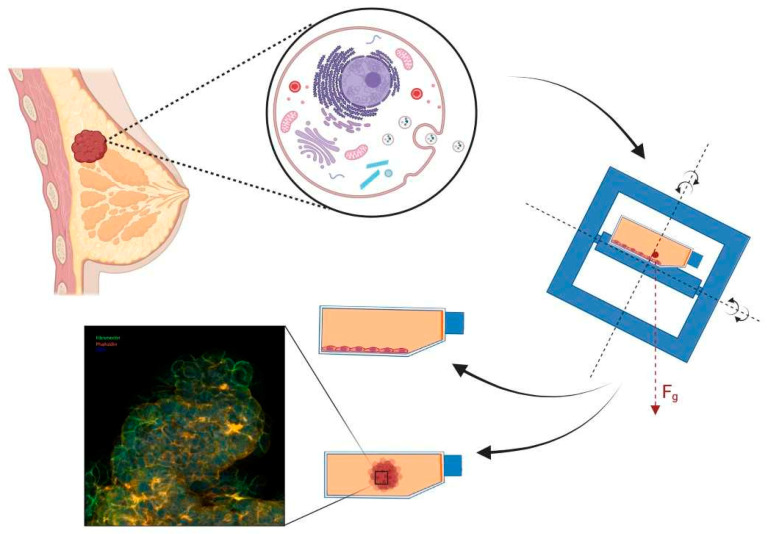
Graphical overview of the phenotypical changes of breast cancer cells after exposure to s-µ*g*. Part of the MCF-7 breast cancer cultures continued to grow in a monolayer, others changed their morphology into 3D spheroids. Confocal microscopy of an MCF-7 multicellular spheroid formed under conditions of s-µ*g*. It shows expression of fibronectin and phalloidin (F-actin). The nuclei are stained with DAPI (4′,6-diamidino-2-phenylindole). Created with BioRender.com (nr. OZ267JA2VC).

A second study investigated the supernatants of MCF-7 breast cancer cells, which were harvested following 5 d or 10 d of RPM-exposure [190]. The authors measured a substantial elevation of released vesicles following cultivation on the RPM (Figure 4). Moreover, the distribution of subpopulations concerning the surface protein expression was also changed; the minimal differences between both time points hint at an early adaption [190]. These two publications gave further insight into the mechanisms of tumor expansion, tumor microenvironments, and the preparation of the metastatic niche and metastasis.

Most of the studies investigated gene expression changes of BC cells cultured under µ*g* conditions. MCF-7 BC cells exposed to real µ*g* showed an early upregulation of *KRT8*, *RDX*, *TIMP1*, *CXCL8* mRNAs, and a downregulation of VCL after the first parabola of a PF [191]. In addition, MDA-MB-231 BC cells exposed to PF maneuvers revealed an early upregulation of *ICAM1*, *CD44*, and *ERK1* mRNAs after the first parabola (P1) and a delayed upregulation of *NFKB1*, *NFKBIA*, *NFKBIB*, and *FAK1* after the last parabola (P31). The *PRKCA*, *RAF1*, *BA*X mRNAs were not changed and cleaved caspase-3 was not detectable in MDA-MB-231 BC cells exposed to PF maneuvers [192].

Five-d-old adenocarcinoma, CRL2351 MCS were formed by RPM-exposure. MCS and AD cells showed an elevated *VIM*, *RHOA*, *MAPK1*, and *BRCA1* gene expression, whereas ERBB2 was only significantly upregulated in MCS. *RAB27A* showed no significant alteration in gene expression [193]. In contrast, after 24 h, the BRCA1 mRNA was upregulated in AD cells, and KRAS was reduced in AD cells compared with 1*g* controls. *VCAM1* was significantly upregulated in AD cells and MCS. In comparison to a five-d RPM-exposition, the 24 h CRL2351 AD and MCS BC cells exerted a significantly downregulated *VIM* expression. There was no significant alteration in the expression of *MAPK1*, *MMP13*, *PTEN*, and *TP53* [194].

µ*g* influenced various biological processes. These changes occurred time-dependently and were dependent on the tumor type. In general, µ*g* had impact on among others survival [195], apoptosis, adhesion, focal adhesion, migration, growth, the cytoskeleton, extracellular matrix, growth factors, several signaling pathways such as PAM, MAPK, and VEGF signaling [196,197,198].

MCF-7 and MDA-MB-231 BC cells were exposed for 14 d to an RPM. Both cell types grew in form of two phenotypes: (1) as an adherent 2D monolayer and (2) as 3D multicellular spheroids [196]. The *ERK1*, *AKT1*, *MAPK14*, *EGFR*, *CTNNA1*, *CTNNB1*, *ITGB1*, *COL4A5*, *ACTB*, and *TUBB* mRNAs of spheroids were differentially regulated in the MCF-7 and MDA-MB-231 cells [196]. Bioinformatics demonstrated a positive association between the real metastatic microtumor environment and 3D spheroids with respect to the cytoskeleton, the extracellular matrix, focal adhesion and EGF/MAP signaling, dependent on the BC type [196].

Overall, the available literature provides novel knowledge regarding the growth behavior of BCCs and molecular biological changes in r-µ*g* and s-µ*g*.

### 3.7. Gynecologic Cancer

Gynecological cancer was responsible for 604,000 new cases and 342,000 cancer deaths worldwide in 2020. This cancer is the fourth most commonly diagnosed tumor as well as the fourth greatest cause of cancer death in females [46].

Gynecologic cancer comprises tumors starting in the female reproductive organs. The five main types are: vaginal, vulvar, uterine, cervical, and ovarian cancer. The NASA-developed RWV has been applied to study the growth and spread of ovarian cancer cells. The researchers investigated biological and chemotherapeutic agents, gene expression changes, and genetic alterations of cancer cells [199]. In addition, NASA supported biomedical research related to women’s health. It also covered the influence of long-term radiation exposure on the endometrium [199]. A recent study reported about 3D cultures of ovarian follicles in a rotating cell culture system [200]. The authors showed that µ*g* did not influence survival or growth of the follicles but decreased the quality of oocytes released from the cultured follicles. Quality improvements of the oocytes could be achieved by GDF9 and NADPH-oxidase supplementation [200].

A further publication focused on the impact of s-µ*g* (36 h) on the process of proliferation and in vitro decidualization using primary human endometrial stromal cells (eSCs). A decrease in proliferation and migration of eSCs without induction of cell death and changes in cell cycle progression was found [201]. µ*g*-exposure of the eSCs inhibited the decidualization by decreasing proliferation and migration through Akt/MMP and FOXO3a/autophagic flux [201]. An early study demonstrated the suitability of the RWV to investigate the regulatory factors that govern tumor angiogenesis. The authors studied endothelial-tumor epithelial cell interactions in human cervical cancers [202]. Furthermore, Jackson et al. [203] used the RWV to investigate collagen-coated microbeads as a growth scaffold and demonstrated the engineering of 3D cellular aggregates. They applied epithelial cells derived from HPV-positive and HPV-negative oral and cervical tissues. The authors used this model among others to characterize the gene expression pattern of these cells by qPCR [203].

Only three publications published the impact of µ*g* on gene expression and protein alterations of cancer cells derived from gynecologic cancers.

Human cervical carcinoma CaSki cells were flown on the Chinese Shenzhou-IV space mission. Guo et al. [204] studied the cell morphology and proliferation. Growth changes and morphological alterations were detected in the 48A9 CaSki cell flight group. MTT and soft agar assay showed that 48A9 CaSki cells grew slowly compared to corresponding ground controls. In addition, the authors demonstrated differentially expressed genes (DEGs) between flight and ground groups. These DEGs included genes belonging to the cytoskeleton, apoptosis, differentiation, signal transduction, DNA repair, protein synthesis, substance metabolism, and antigen presentation [204].

A new cheap and robust customized small-sized clinostat (CS clinostat) was recently designed as well as fitting gas-permeable polydimethylsiloxane culture dishes [205]. Ovarian cancer cells (OV-90, TOV-21G, and Caov-3) were tested and showed a significant reduction in caveolin-1 protein content [205].

Przystupski et al. [206] used short-term clinorotation (2 h) to study the cisplatin sensitivity of the drug-resistant human ovarian cancer cells SKOV-3. µ*g* exposure influenced morphology (signs of apoptosis) as well as drug efficiency. The authors showed an increase in apoptotic cells and G0/G1 cell cycle arrest after clinorotation and treatment with cisplatin in comparison to the static control cells. Moreover, the proliferation rate and migration were altered after the clinostat-exposure. These results demonstrate that µ*g* influenced the mechanisms responsible for cisplatin resistance of the SKOV-3 cells resulting in a higher drug sensitivity [206].

Taken together, omics studies of gynecologic cancers and normal cells from the gynecologic organs are still lacking but are nevertheless of high importance. Space is an extreme environment for our life and cosmic radiation and µ*g* are affecting the health of all space travelers. Women are known to have a higher radiation-induced cancer risk and incidence for example for breast and ovarian cancers, among others [207]. Therefore, space research focusing on gynecologic cancer types is necessary to increase our knowledge about the risks for women in space and to find effective countermeasures as well as to evaluate the cellular and molecular changes detected in cells exposed to µ*g* conditions.

### 3.8. Gastrointestinal Cancer

This subchapter has encompassed the different omics data available from the research of gastrointestinal cancers involving colorectal cancer cells, gastric cancer cells, liver cancer, and pancreatic cancer cells cultured under both r- and s-µ*g* conditions. All these cancers are very common and leading causes of cancer-related deaths worldwide. To understand the regulatory mechanisms governing the alterations in gastrointestinal tumors under µ*g* conditions, a comprehensive analysis of various omics layers, such as genomics, metabolomics, transcriptomics, and proteomics studies are imperative. These layers operate independently while maintaining interrelated roles in this context.

So far, the human MIP-101 cell line was studied under both r- and s-µ*g* conditions. The cell lines studied under s-µ*g* conditions were HCT116 colon cancer cells, DLD1, SW620, LS180 colorectal cancer cells, the colonic fibroblast cell line HT-29, HT-29KM, HT-29KM CCL 188 KM-12c, HGC-27 (gastric cancer). Furthermore, the resistant and sensitive human Gastric Cancer Cells (EPG85–257 RDB and EPG85–257 P), the liver cancer HepG2 cells, human biliary tree stem/progenitor cells (hBTSCs), the pancreatic cancer cells NOR-P1, PaCa44, and Caco-2 were investigated.

As our focus is only on omics data, in this review we are discussing the data obtained from HCT116, MIP-101, DLD1, LS180 colorectal cancer cells, HGC-27 gastric cancer cells, human resistant and sensitive gastric cancer cells, liver cancer HepG2 cells, pancreatic cancer (PaCa44), and Caco-2 cells.

Although not many gastrointestinal cancer cells have been studied under r-µ*g* on the ISS, Reynolds et al. [208] conducted a study on astronauts from the United States during the period from 1959 to 2017, which explored various pertinent aspects such as cancer-specific mortality rates, cancer incidence rates, and cancer case-fatality ratios and found that colon cancer exhibited a noteworthy (though statistically not significant) decrease in both its incidence and mortality rates among astronauts in contrast to general US population. The observed reduction in colon cancer cases among astronauts could be explained by healthy lifestyle choices and tailored screening protocols among astronauts.

Arun et al. [209] studied the effect of s-µ*g* for a period of 72 h on cancer stem cells (CSCs) and HCT116 cell using a rotary cell culture system (RCCS). The study of HCT116 cells under s-µ*g* and the 3D cell cultures provided insights into the regulation of autophagy, YAP signaling, and the absence of Wnt activation. These findings contribute to our understanding of cellular responses to different environmental factors and their impact on cellular processes and signaling pathways. The proteomic data in respect to progression of autophagy is marked by the conversion of LC3B-I into LC3B-II, which occurs through the conjugation of phosphatidylethanol. The LC3B-II upregulation was observed in both the 3D culture and s-*µg* conditions. Autophagosome Maturation Elements ATG5 and ATG12 were upregulated in both 3D and s-µ*g* conditions. Proteins involved in autophagosome formation, including ATG7, ATG16L1, and Beclin1, were downregulated in the 3D condition and mildly elevated in s-µ*g*. In the 3D condition, AKT (protein kinase B) was upregulated, suggesting obstruction in the upstream signal for autophagosome formation. This upregulation was not observed in s-µ*g*. FOXO3 (forkhead box O3) and PTEN (phosphatase and tensin homolog) were upregulated in both the 3D and s-µ*g* conditions, indicating increased autophagy activation signaling. Nuclear localization of YAP1 (Yes-associated protein 1) resulted in downstream signaling with a significant increase in the Yamanaka factors, including OCT4A, SOX2, Nanog, and NKx-2.5. This suggests that YAP1 plays a role in regulating these factors. Nuclear localization of β-catenin, a key component of the Wnt signaling pathway, was completely absent in the experimental conditions, indicating minimal Wnt activation in HCT116 cells.

Jessup et al. [210] conducted a study on Human MIP-101 poorly differentiated colorectal carcinoma cells, where the cells were cultured for 6 d in a full growth medium in three different conditions. Monolayers on Teflon^®^-coated nonadherent surfaces in a static 3D setting, in rotating 3D cultures in a µ*g*-environment in low-earth orbit (3D μ*g*) and in rotating 3D cultures under the influence of standard unit gravity on the ground (3D 1*g*). The results suggested that the culture conditions, particularly the presence or absence of rotation and µ*g*, have a significant impact on the expression of growth factors and CEA-related proteins in MIP-101 cells. This information provides valuable insights into proteomics on cellular responses under different gravitational and growth conditions. Cells growing in static 3D conditions exhibited the highest expression of EGF-R, TGF-α, and TGF-β1. Cells in monolayer cultures also had relatively high expression of these growth factors and EGF-R, though slightly lower than static 3D cultures. In contrast, cells in rotated cultures expressed significantly less TGF-α, TGF-β1, and EGF-R compared to the other culture conditions. Rotated cultures, whether in unit gravity or µ*g*, did not show significant differences in the expression of growth factors during the initial period of rotation (day 2–5). However, expressions of EGF-R, TGF-α, and TGF-β1 appeared to be higher in non-rotated (static) cultures compared to rotated cultures during this period. When rotated cultures were allowed to remain static after day 6, there was an increase in the expression of EGF-R, TGF-α, and TGF-β1. CEA expression was found to be upregulated in rotated µ*g* cultures compared to cultures rotated in unit gravity. This upregulation included not only the 180-kDa CEA but also the 160-kDa biliary glycoprotein (BGP) and the 55-kDa form of NCA. Overall rotated 3D µ*g* cultures expressed more CEA and related molecules than rotated 3D unit gravity culture in MIP-101 cells.

Vidyasekar et al. [111] conducted a study involving the DLD1 colorectal and MOLT-4 lymphoblast leukemic cells under µ*g* conditions, using the RCCS bioreactor. The result showed the upregulation of *STAT3*, *DLL1* (Notch signaling pathway) and *HEY1* and the downregulation of the retinoblastoma gene (*RB1*) in DLD-1 cells under µ*g* and its potential role in stress-induced premature senescence (SIPS). The other important findings from this study were dysregulation of *EIF2C1* and *EIF2C3* (components of RNA-induced silencing complex), *TLE3* (WD40 repeat domain protein), *RPS27A* (ribosomal protein), *APH1B*, *PSEN2*, and *PSENEN* (involved in γ-secretase complex and Alzheimer’s disease), *TLE1*, *TLE2*, and *FBXW7* (WD40 repeat domain proteins), *SMAD1*, *SMAD2*, and *SMAD3* (involved in microRNA maturation) *DROSHA* and *DICER* (core components of miRNA processing). Dysregulation of microRNA host genes, including *MIR17HG* and MIR21HG. These findings suggest differential gene expression patterns in response to microgravity in the DLD1 line. Additionally, the miR-22 host gene appeared to play a significant role in DLD-1 cells under µ*g*, with potential implications for cancer progression and cell fate determination.

Arun et al. [211] described the effect of s-µ*g* using Rotational Cell Culture System-High Aspect Ratio Vessel (RCCS-HARV) on DLD1, HCT116 and SW620 cells. The gene expression analysis of DLD1 cells showed the following findings. The mRNA levels of cell cycle genes *CDK1*, *CDK2*, *CCNB1*, and *CCNE1* diminished in DLD1 cells subjected to s-µg. In addition, the cell cycle inhibitors *CDKN2B* (p1INK4b) and *CDKN2D* (p16INK4d) were significantly higher in s-µ*g*, with *CDKN2D* expression maintained and *CDKN2B* downregulated. Western blot analysis revealed high expression levels of stress response elements such as p38 MAPK, STAT3, cell–cell contact protein E-cadherin, and MnSOD in s-µ*g* compared to control cells [211]. AKT-related pathways were significantly modified in the microarray analysis, suggesting possible canonical/non-canonical intervention of the pathway. PTEN, a major inhibitor of AKT activation, was upregulated in s-µ*g*. Protein levels of AKT, as well as phosphorylated forms pAKTs473 and pAKTt308, were diminished in s-µ*g*. The phosphorylated form of GSK-3β, a marker for the progression of the AKT pathway, was also diminished. PTEN, its phosphorylation at serine 380, and FOXO3 were upregulated during s-µ*g* [211]. Prolonged culture under static conditions showed upregulation of AKT, phosphorylated forms, and FOXO3, which was significant compared to control cells. Notably, genes such as *MIR17HG*, *MIR22HG*, *MIR21HG*, *CD44*, *JUNB*, *MYC*, and *CD117* demonstrated significant alterations which shed light on the disruption of these critical genetic elements in response to µ*g*. The protooncogene *MYC* was shown to be upregulated and *CD117* was downregulated in colorectal cancer. The mRNA levels of autophagy-controlling genes indicated that genes involved in autophagosome formation were high in s-µ*g* (*ATG4B*, *ATG7*, *ATG16L1*, *Beclin1*, and *LC3B*), while genes involved in autophagosome maturation (*ATG5* and *ATG12*) were downregulated [211].

These findings suggest that the FOXO3-dependent mechanism of cell destruction and autophagy is activated but not matured in s-µ*g*. The results highlight the role of the PTEN/FOXO3/AKT axis in the control of cell fate during and after s-µ*g*. Cell death under s-µ*g* is mediated through upregulation of CDK inhibitors CDKN2B and CDKN2D through tumor suppressors FOXO3 and PTEN. The gene expression analysis on DLD1 cells showed upregulation of tumor suppressors *PTEN* and *FOXO3*, leading to *AKT* downregulation and further induction of apoptosis through upregulation of CDK inhibitors *CDKN2B* and *CDKN2D.* These findings suggest complex molecular interactions involving cell cycle regulation, stress response, and autophagy pathways in response to s-µ*g* [211].

In another study conducted by Smit et al. [212], LS180 colorectal cancer cells were cultured as 3D sodium alginate encapsulated spheroids in clinostat bioreactors for a period of 20 d. Authors reported that over the first 10 d of culturing, there was a steady increase in intracellular ATP content per μ*g* protein. However, a temporary decrease occurred from day 10 to day 13, which was associated with the handling of spheroids (transfer to another bioreactor). Subsequently, the spheroids began to recover, and there was a significant increase in ATP content on days 17 and 20 compared to the initial ATP content on day 0 (*p* < 0.001). Extracellular AK release per μ*g* protein remained low for the first 6 d. A steady increase was observed until day 10, indicating cell death, possibly due to competition for nutrient resources. The reduction in the number of spheroids on day 10 led to a decrease in extracellular AK release from day 10 to 17, followed by a slight increase on day 20. Moreover, the study tracked glucose consumption, with fresh medium containing 6.8 mmol/L glucose provided every 48 h [212]. Glucose levels in the spent medium decreased rapidly, falling below 1.1 mmol/L after three days, and being undetectable between days 6 to 10. Even after the reduction in spheroid density on days 10 and 15, glucose depletion in the spent culture medium persisted [212]. Treatment with paclitaxel led to an increase in P-gp efflux pump gene expression in spheroids after 96 h of treatment. This suggested that the spheroids responded to the treatment by upregulating the expression of P-glycoprotein, which was associated with drug resistance. The study highlighted the potential for drug resistance, as evidenced by increased P-gp gene expression in response to paclitaxel treatment [212].

A metabolomics study was performed by Chen et al. [213], who cultured HGC-27 stomach cancer cells in the RCCS bioreactor. In their study, Chen et al. revealed notable alterations in metabolic expression under s-µ*g* conditions in compared to a normal gravity control group. Specifically, they observed significant upregulation of phosphatidyl choline, phosphatidyl ethanolamine, arachidonic acid, and sphinganine, while phosphatidyl serine, sphingomyelin, phosphatidic acid, creatine, L-proline, pantothenic acid, adenosine triphosphate, oxidized glutathione, and adenosine triphosphate exhibited significant downregulation. This study underscores the profound metabolic changes experienced by HGC-27 gastric cancer cells in a microgravity environment.

Rembiałkowska et al. [214] demonstrated that s-µ*g* conditions could alter the expression of MDR (Multi-Drug Resistance) genes in gastric cancer cells. Specifically, they observed that both µ*g* alone and µ*g* with the addition of doxorubicin (DOX) treatments resulted in reduced the expression of the *ABCB1* gene in EPG85-257 RDB cells compared to normal gravity (1*g*) conditions. For the sensitive cell line, exposure to 20 rpm centrifugation under 1*g* conditions led to an increase in the expression of the *ABCG2* gene. However, when these sensitive cells were exposed to s-µ*g* conditions with DOX for 72 h, there was a notable decrease in the expression of both *ABCB1* and *ABCG2* genes when compared to cells exposed to 1*g*. In the resistant cell line, a decrease in the expression of *ABCG2* and *LRP1* was observed in cells exposed to µ*g* when compared to the control cells. This study underscored the complex and distinct effects of microgravity on *MDR* gene expression in both sensitive and resistant gastric cancer cells, shedding light on the potential impact of µ*g* on drug resistance mechanisms.

Metabolomics data from the pancreatic cancer cell line PaCa44 were published by Masini et al. [215]. They investigated the effects of long-term exposure to s-µ*g* using the RPM on PaCa-44 cells and performed proteomic, lipidomic and transcriptomic analyses after 1 d, 7 d, and 9 d. For the PaCa-44 cell line, glycolysis was initially downregulated after 24 h in s-µ*g* but was upregulated after 7 d, and this upregulation continued to the 9th day. Extracellular lactate dehydrogenase (LDH) levels indicated a reduction after 1 d in s-µ*g* and an increase on the 7th and 9th day. After 9 d of s-µ*g*-exposure, there was upregulation of various proteins involved in metabolic-related pathways, including autophagy, mitochondrial activity, the TCA cycle, and the Pentose phosphate pathway.

The authors also focused on eIF2 signaling, the most enriched pathway modulated by s-µ*g*. It was inhibited after 24 h and remained downregulated for up to 9 d [215]. The phosphatidylinositol-3-kinase/Akt (PI3k/Akt) pathway was upregulated on the 1st and 7th day of s-µ*g*-exposure but downregulated on the 9th day. Upregulation of NFκB signaling, indicating increased activity of this signaling pathway in s-µ*g*.

In addition, the upregulation of stemness-related proteins, including EpCAM, ALDH1A3, ALDHA3A2, S100A4, ROHA, and ITGA3, suggested a shift towards a more stem-like and aggressive cellular phenotype. Upregulation of glycolysis-related proteins after 7 d in s-µ*g*, with a positive logFC for ENO1, GAPDH, ALDOA, PGAM1, TPI1, and PKMI proteins were observed. This indicated an increase in glycolytic activity. The upregulation of LDHA, GAPDH, PKM, and SLC2A1 proteins in the glycolysis pathway on the 9th day in s-µ*g* was also found. A reduction of extracellular lactate dehydrogenase (LDH) levels after 1st day in s-µ*g* indicated the changes in lactate production. Initially, HIF-1α signaling was inhibited at 24 h and 7 d in s-µ*g*, but it was upregulated on the 9th day, suggesting a dynamic regulation of this pathway. The proteomic data highlighted significant changes in protein expression related to various cellular pathways, including NFκB signaling, stemness, glycolysis, and HIF-1α signaling in response to s-µ*g* exposure. These changes demonstrated a complex and dynamic response of the cells to altered gravity conditions over time [215].

Moreover, the authors provided also lipidomic data regarding the PaCa44 cells exposed to s-µ*g* [215]. A total of 447 lipids from 18 different lipid classes were identified in PaCa-44 cells. Triacylglycerols (TGs) were the most abundant lipid class in these cells. Monoacylglycerols were not detected, while only a few diacylglycerols were found, possibly due to their re-esterification into TGs [215]. Hierarchical clustering heat-map analysis revealed specific s-µ*g*-induced lipidomic profiles at different time points. Total TG levels decreased at 1 d and 7 d in s-µ*g* but increased at 9 d. Total lysophosphatidylcholines (LPCs), lysophosphatidylethanolamine (LPEs), and polyunsaturated fatty acids (FAs) increased at 1 d and 7 d in s-µ*g* but decreased at 9 d [215].

A proteomic study was published by La Barbera et al. [216] who reported that 38 and 26 proteins were differently regulated by s-µ*g* after 48 h and 72 h (2D clinostat). After a 48 h s-µ*g* exposure, certain proteins associated with ATP synthesis and mitochondrial functions (e.g., MTFR2, MT-ATP8) were reduced. This demonstrated a potential disruption in cellular energy production in s-µ*g* conditions. Notably, ubiquitin-associated proteins (UBAP1, UBR3) showed an overexpression after both 48 h and 72 h of s-µ*g* exposure. This suggests an upregulation of protein degradation pathways in response to microgravitational stress. Proteins related to intestine morphological organization and peptide transport, such as cadherin 17 (a calcium-dependent cell adhesion protein), were found to be overexpressed. This might indicate a compensatory response to maintain intestinal integrity in µ*g*. CDK2, a critical regulator of cell-cycle and epigenetic processes, was significantly downregulated after 48 h of s-µ*g* exposure, implying potential disruptions in cell-cycle regulation and epigenetic modifications in s-µ*g* conditions. Heterogeneous nuclear ribonucleoprotein D-like (HNRNPDL), specifically its subunit DnaJ homolog subfamily C member 5, was downregulated after 72 h of s-µ*g*-exposure, indicating altered RNA processing and splicing in response to prolonged microgravity conditions. A significant finding was the lower basal activation of NFκB in s-µ*g* conditions. This suggests that the immune response and inflammatory signaling might be suppressed in s-µ*g*. Another important observation was that µ*g* did not alter the CYP27A expression or its transcriptional regulation in the Caco-2 cell model, proposing that certain cellular functions could remain unaffected by s-µ*g*.

Khaoustov et al. [217] utilized the RCCS to simulate a µ*g*-environment and analyzed the gene expression in a hepatoblastoma cell line (HepG2) during the early stages of 3D cell assembly. The key genomics changes were as follows: The preliminary analysis of 4673 human genes revealed significant alterations in the expression of 95 genes. Out of these 95 genes, 85 genes were found to be upregulated, while 10 genes were downregulated when cells were cultured in the RCCS, replicating a microgravity-like environment. Notably, 10 genes, including serine hydroxymethyltransferase 2 (mitochondrial), insulin receptor, apolipoprotein E, and peptidylprolyl isomerase F (cyclophilin F), showed a more than 10-fold increase in expression compared to the control. The maximum increase observed was 80-fold. An additional 40 genes were upregulated more than threefold. Some of the upregulated genes, such as *KIAA0073*, *EST* (Hs.55153), and genomic sequences, had unknown functions. The study also revealed changes in the expression of specific transcriptional factors, including the Wilms tumor I protein, in s-µ*g* conditions. In another study from Chang et al. [218] the human liver hepatocellular carcinoma cell line (HepG2) was cultured for 72 h in a RWV to simulate µ*g*. In addition, the cells were cultured as monolayers on tissue culture dishes (TCDs). Afterwards, a global gene expression analysis was performed. RWV-spheroids showed an upregulation of metabolic (*CYP1A1*, *AKR1C1*, *EPHX1*, *LTB4DH*, *LDLR*, *HMGCR*) and synthetic genes *(GSTA1*, *GCLM*, *ALB*, *ATP5I*, *NDUFA3*), suggesting functional differences.

Finally, Clement et al. [219] conducted a comprehensive analysis of global gene expression profiles in a human liver cell line exposed to the RCCS for varying durations (1 d, 3 d, and 4 d). The authors used Agilent 22k human oligo DNA microarrays. The key Genomics data are as follows: A total of 139 genes were identified whose mRNA levels were significantly altered (with a *p*-value ≤ 0.01) in response to µ*g* exposures. Several genes associated with GTP binding activities exhibited a significant downregulation, indicating a decrease in their expression levels under microgravity conditions. Conversely, genes with ATP-binding activities appeared to be upregulated in response to µ*g*, suggesting an increase in the expression of these genes. Genes responsible for lipid transporter activities, such as *APOA1*, *APOA2*, and *APOB*, were significantly downregulated. This indicated a decrease in the expression of genes associated with lipid transport. Translation initiation factor activities, particularly ElF3S8 and EIF4AI, were also significantly lower which pointed to a reduction in the expression of genes involved in translation initiation in the presence of µ*g*.

### 3.9. Lung Cancer

Lung cancer is among the most common malignant diseases worldwide. In 2020, for both sexes combined it was estimated that it was responsible for a total of 2,201,419 new cancer cases (2nd place after breast cancer) and 1,796,144 deaths (1st place), representing a severe global health issue [46,220]. The mean 5-year survival rate for lung cancer is about 20% but falls rapidly for more advanced stages. In case of metastatic disease, which represent almost 60% of the cases, it is only about 5% [221]. Preventing metastases should therefore be one of the main goals in lung cancer treatment. Furthermore, 3D cell culture under (s-)µ*g* might shed some light onto possible novel metastasis-related therapy approaches.

Chang et al. exposed cells from the human lung adenocarcinoma-derived line A549 to a horizontal MG-3 clinostat for 72 h. They found that under s-µ*g*, both invasion and migration were significantly reduced to about 50% compared to 1*g* controls. Semiquantitative PCR analyses showed that both *MMP2* and *MKI67* gene expressions were downregulated in s-µ*g*, although not significantly. Interestingly, cells overexpressing MMP-2 protein showed significantly increased migration and invasion on the clinostat. The authors concluded that s-µ*g* might reduce the metastatic potential of A549 cells by mediating MKI67 and MMP-2 [222].

Similar experiments on A549 and lung squamous cell carcinoma H1703 cells were conducted by Chung et al. They cultured both cell lines on a 3D clinostat for 36 h. Both cell lines reacted differently to the s-µ*g* stimulus. While the proliferation rate of A549 did not change, it was significantly reduced in H1703 cells. Migration was increased in both cell lines, but changes were statistically significant only in H1703 cells. qPCR analyses of gene expression profiles of *MMP2*, *MMP9*, *TIMP1*, and *TIMP2* revealed a significant downregulation of all genes in A549 cells under s-µ*g*. Interestingly, similar effects could not be found on the protein level. In H1703 cells, *MMP2* and *MMP9* genes were significantly downregulated on the clinostat, whereas the slight reduction in *TIMP1* and *TIMP2* gene expression was not significant. Of note, the protein expression levels of MMP-2, MMP-9, and TIMP-1 seemed to increase after 24 h, but to decrease after 36 h of s-µ*g* exposure, always remaining lower than those in control cells. However, these results did not show statistical significance. Overall, s-µ*g* seemed to exert an effect on proliferation and migration that is different for different lung cancer cell lines [223].

The same cells were also used by Ahn et al.; however, they employed a self-made device that aimed to simulate µ*g* by culturing cells on a floating membrane inside a medium-filled chamber for up to 48 h. After 24 h both cell lines exhibited significantly increased gene expression of *MMP2*, *MMP9*, *TIMP1*, and *TIMP2* compared to 1*g* controls. For H1703 cells, this remained unchanged after 48 h, while at this time, A549 only showed a significant upregulation of *MMP2*. Interestingly, there were some distinct differences in cell lines regarding MMP-2 and MMP-3 protein expression. While In A549 cells both MMP-2 and MMP-3 proteins were significantly elevated compared to 1*g* controls after 24 h and 48 h, H1703 reacted diametrically differently. Here the authors found a significant downregulation of both proteins compared to 1*g* controls, with MMP-2 being undetectable after 48 h in both 1*g* and s-µ*g* samples [224].

Degan et al. exposed A459 cells to a Random Positioning Machine for up to 48 h. Beside observing the generation of polynucleated cells, cell cycle imbalance, growth inhibition, morphological abnormalities, and highly damaged mitochondria, they also performed a global MiRNA analysis. In total, the authors found 6 downregulated miRNAs (*hsa-miR-16-5p*, *hsa-miR-194-5p*, *hsa-miR-20a-5p*, *hsa-miR-221-3p*, *hsa-miR-30c-5p*, *hsa-miR-34a-5p*), and four upregulated miRNAs (*hsa-let-7b-5p*, *hsa-miR-107*, *hsa-miR-193b-3p*, *hsa-miR-29a-3p*). They then selected the most representative gene set which was associated with these miRNAs and identified a total of 13 up- and 38 downregulated genes (with 6 genes occurring in both groups). This revealed that the differentially expressed miRNAs are mainly involved in cell cycle regulation (via P53, CDKN, E2F), apoptosis (via BCL2, BIRC5), and stress response (via FOS, MAPK). The authors suggested that mitochondria are sensitive for µ*g* and the resulting mitochondrial damage might lead to cell cycle disturbances via modulation by differentially expressed miRNAs [225].

Baghoum et al. chose a combined approach using publicly available gene expression data for in silico analyses and their own experimental data from A549 cell cultured on a Rotating Wall Vessel system for 24 h, 48 h, and 72 h. For this, the authors chose two datasets from the Gene Expression Omnibus (GEO) database (GSE78210 and GSE36931), covering gene expression profiles for two different human lung cancer cell lines, A549 and Colo699, cultivated in 2D and 3D cell culture conditions. Of the total 251 identified differentially expressed genes, 13 were found to be present in both datasets in A549 cells. These candidate genes comprised *AZGP1*, *CFB*, *NOX1*, *VTCN1*, *AGR3*, *GDA*, *TCN1*, *CST1*, *F5*, *CEACAM6*, *BPIFB1*, *FCGBP*, and *BPIFA1*. Afterwards, the authors compared overall survival of lung adenocarcinoma patients with strong vs. medium/low candidate gene expression and found high mRNA expression levels of *NOX1*, *GDA*, *TCN1*, and *BPIFA1* to be significantly associated with poor prognosis, while high mRNA expression levels of *FCGBP* were connected to a longer overall survival. No significant correlations were found for *AZGP1*, *CFB*, *VTCN1*, *AGR3*, *CEACAM6*, *F5*, and *BPIFB1*. For further validation, a qPCR analysis of A459 cell cultured in s-µ*g* compared to 1*g* controls was conducted using the identified target genes. *FCGBP* was significantly upregulated after 46 and 72 h, and *CFB*, *F5*, and *BPIFB1* were significantly elevated after 72 h. Lastly, it was found that s-µ*g* significantly reduced cell viability after 48 and 72 h. To account for this finding, key epithelial-to-mesenchymal transition (EMT) markers (*CDH1*, *CDH2*, *TJP1*, *SNAI1*, *MMP2*, and *MMP9*) were analyzed by qPCR. A significant increase in *CDH1* as well as significant decreases in *CDH2* and *MMP2* gene expression und s-µ*g* indicated an induction of a mesenchymal–epithelial transition (MET) phenotype in the A549 cells, suppressing cancer progression [226].

Dietz et al. used cells from the squamous, non-small cell lung cancer line CRL-5889 and exposed them to up to 96 h of s-µ*g* on the RPM. After about 48 h some cells in s-µ*g* detached from the substrate and formed spheroids, which increased in size over time. In s-µ*g*, cell showed an alteration of their actin cytoskeleton, shifting from the usual parallel, longitudinal arrangement of filaments to accumulations of actin near the membrane and a more amorphous, disordered appearance in the cytosol. Compared to both 1*g* control as well as adherent cells in s-µ*g* (AD), cell death as detected by TUNEL was significantly increased in spheroids. Interestingly, only AD cell showed differentially increased expression of genes involved in cell death/apoptosis, such as *TP53*, *CDKN2A*, *PTEN*, and *RB1*. Overall, the authors could show that s-µ*g* mediates cell adherence, increases cell death, and leads to the upregulation of tumor suppressor genes in human lung cancer cells [227].

### 3.10. The Potential Biases or Limitations of This Review

The interpretation of omics data from studies conducted under µg conditions may be subject to biases related to the complexity of analyzing biological responses to µg and the potential for a more subjective interpretation of results. Furthermore, the findings and conclusions drawn from omics studies of tumor cells cultured under µ*g* conditions may have limitations in generalizing the results to broader cancer biology contexts or clinical applications.

## 4. Conclusions

Exploration of the Universe has become reality. Since the 1990s, a large number of publications have appeared reporting changes in gene expression, protein synthesis and secretion in cells, tissues and animals exposed to r- and s-µ*g* [20,32,44,228,229,230,231,232,233,234,235,236,237]. As a result, we are expanding our knowledge more and more about the stay and its effects on humans, animals, microorganisms, bacteria, and cells in space. Simulation experiments and tests in real microgravity (parabolic flights, sounding rocket missions, American Shuttle flights, Russian long-term Foton or Bion and Chinese Shenzhou missions, short- and long-term stays on space stations (Mir, ISS, Chinese space station)) have already increased our knowledge about the dangers of microgravity, cosmic radiation, and other stress factors occurring during and after space missions [238,239,240,241,242,243,244,245,246,247,248,249,250,251,252].

**Figure 5:**
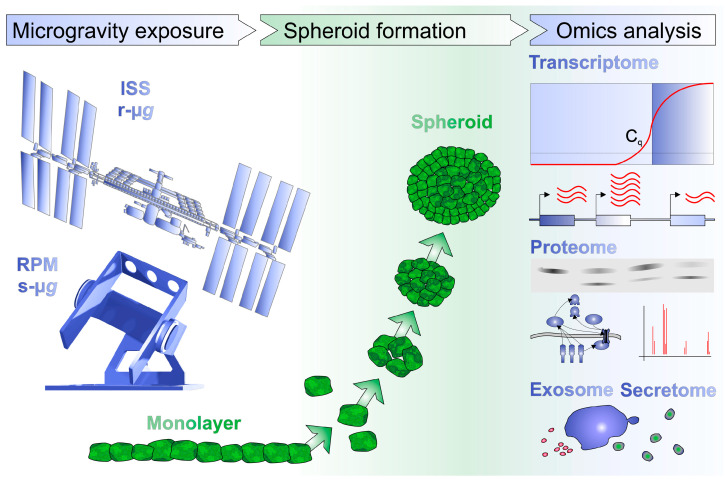
Usual features of an omics-based tumor study under microgravity. Tumor cells exposed to real or simulated microgravity have changes in cell interaction compared to the 2D monolayer which induce physiological and morphological changes with transcriptional, proteomic, exosomic, or secretomic consequences.

This review discusses the available literature about omics studies of cancer cells exposed to conditions of real and simulated weightlessness with focus on the transcriptome, proteome, secretome and metabolome (Figure 5). For an overview of the different types of cancer in tabular form, please see Appendix A. Various studies reported the effects of microgravity on human tumor cells, their adaptation mechanisms, and related cellular alterations. In particular, microgravity-exposed cancer cells revealed alterations in the cytoskeleton, the extracellular matrix, adhesion, migration, proliferation, differentiation, focal adhesion molecules, growth, survival, apoptosis, and cell signaling.

New molecular biological methods such as omics investigations together with advances in Bioinformatics are able to clearly characterize the risks of space travelers, to protect their health and help to develop adequate countermeasures. This knowledge obtained from space research also supports translational regenerative medicine and in particular cancer research on Earth.

It is important for the analysis of data from space experiments to distinguish unspecific stress reactions from specific microgravity effects. Therefore, novel studies are often used in silico analyses and study protein–protein interactions. These analyses provide new knowledge about functional relationships, even when epigenetic and transcriptional data (multi-omics) are available. These data can be submitted to the NASA GeneLab, an open repository for space biology data. Students, physicians, and specialists can use this platform to get more information about how, for example, human life is affected by space conditions. In addition, they can study the data from cancer cells and other tumor cells cultured in space or on microgravity simulators regarding transcriptomics, genomics, proteomics and metabolomics.

## Data Availability

Not applicable.

## Appendix A

**Table A1:** Overview of omics studies on tumor cells under microgravity conditions.

Cell Line	Omics	Microgravity	Devices/Platforms	Ref.
BRAIN TUMORS				
K562	Transcriptomics: mRNA levels of *STIM1*, *STIM2*, *ORAI1*, and *ORAI* were accessed. S-µ*g* downregulated *ORAI1*. Proteomics: Reduced levels of ORAI1 protein were observed following incubation under µ*g* conditions.	s-µ*g*	2D clinostat	[84]
A-172 and HUVEC	Proteomics: Lower expression of s-µ*g* YAP-1 in A-172 cells. Remodeling of Cadherin junction protein in HUVECs	s-µ*g*	RPM	[85,86]
NSC	Genomics: r-µ*g* alters division of NSC Proteomics: Reduced expression of Tubulin was observed in r-µ*g* exposed NSCs.	r-µ*g*	ISS	[87]
HEMATOLOGICAL DISORDERS				
K562	Metabolomics: Increase production of ROS	s-µ*g*	Clinostat	[100]
Raji cells	Genomics: Reduced DNA repair Proteomics: Reduction of ATM expression Metabolomics: Higher ROS	s-µ*g*	RWV	[101]
U937	Proteomics: No express of 5-Lox	s-µ*g*	RPM	[102]
	Proteomics: Increased expression of PKC, interleukin production significantly reduced	r-µ*g*	Space shuttle flight	[103]
	Proteomics: Decrease in the expression of Actin. Changes in the distribution of vinculin and be-ta-tubulin Upregulation of Hsp70; decrease in proteasome activity	s-µ*g*	RWV	[116]
	Proteomics: Reduction phosphorylation of tyrosine and activation of transcription factor C-Jun. Increase the phosphorylation of p53 protein	s-µ*g* r-µ*g*	2D Clinostat PFC	[113]
	Genomics: Altered in the response of HIF-related genes Transcriptomics: HIF-1α, HIF-1 were transcripts differently regulated. Alteration in transcriptome. Proteomics: Increase the phosphorylation of MEK	r-µ*g*	PFC	[115]
	Proteomics: Increase the expression of ICAM-1	s-µ*g*	2D Clinostat	[112]
	Transcriptomics: Profound alteration in the transcriptome associated with DNA replication. Changes in mRNA processing	r-µ*g*	PFC/TEXUS-49	[117]
	Proteomics: Reduction in PKS translocation	r-µ*g*	STS-81 Space Shuttle mission	[122]
TK6	Transcriptomics: Expression of several miRNAs was changed significantly in the simulated microgravity condition including *miR-150*, *miR-34a*, *miR-423-5p*, *miR-22*, *miR-141*, *miR-618*, and *miR-222*	s-µ*g*	HARV	[110]
	Epigenomics: 3204 DMRs differentially methylated regions, 1286 DMRs associated with hypermethylation (gain of 5mc) and 1918 DMRs associated with hypomethylation (loss of 5mc). Transcriptions start sites of the genes *WBSCR22*, *DOCK6*, *C3*, *DEFB119*, *FXYD6* were hyperhydroxymethylated whereas of the gene *MTRNR2L2* were hypo-hydroxy methylated Transcriptomics: Transcription of genes *TSPAN5*, *SPG20* associated with loss of 5mc is upregulated, whereas genes as *PLIN2*, *MAP3K13*, *FBX01* were downregulated	s-µ*g*	HARV	[109]
	Genomics: Increasing of HPRT mutants and broken chromosome fragment (micronucleus)	s-µ*g*	RWV	[105]
MOLT-4	Genomics: 349 genes were upregulated, and 444 genes were downregulated Epigenomics: Dysregulation of post transcriptional gene silencing machinery Transcriptomics: Significant dysregulation of several microRNA host genes including *MIR17HG*, *MIR21HG. MIR22HG* are upregulated	s-µ*g*	HARV	[111]
Jurkat cells	Proteomics: Enhance the phosphorylation of MAP kinase ERK-1/2, MEK and P38 Inhibition of NFKB	s-µ*g* r-µ*g*	2D Clinostat PFC 8th DLR	[113]
	Proteomics: Different expression of cytoskeletal proteins Vimentin and β-actin were in-creased ß-tubulin reduced and hardly detect-able release of IL-2, TNFα, GM-CSF Metabolomics: Decreased intra-cellular Ca^2+^ and ROS. Decreased glucose and lactate content	s-µ*g*	RPM	[119]
	Genomics: Downregulated of *CD69* gene Proteomics: Increase of Fas/Apo-1 protein Metabolomics: Significant increase in glucose consumption	r-µ*g*	STS-80 STS-95 Space Shuttle missions	[124,133]
	Genomics: Upregulation of *CAPN1* gene. Increase cytosolic DNA fragments Transcriptomics: Increased mRNA significantly (~2-fold) Proteomics: Upregulation µ-Calpain and INF-y significantly reduction of cytokines LIF, IL-4, IL-2 Metabolomics: Increase activity of µ-Calpain (~2-fold). The cytosolic content of cytochrome c increased and lost it in the mitochondria.	s-µ*g*	RCCS	[126]
	Genomics: TSC2 and cell cycle-related genes were upregulated. 2% of 20,000 genes were upregulated. 10 cytoskeletal genes upregulated (Plectin; C-NAP1, Calponin, MLC-2, Ankyrin, and Dynactin) and gene encoding gelsolin downregulated Transcriptomics: mRNA for Plectin, C-NAP1, and Calponin were upregulated Proteomics: Upregulation of CDK6, Tuberin, actionlike protein and EST	r-µ*g*	STS-95 Space Shuttle mission	[130]
	Proteomics: VEGFR-1, VEGFR-2, VEGFR-3 were downregulated	s-µ*g*	RWV	[134]
HL cells (L-540 and HDLM-2)	Genomics: Upregulation of NADPH oxidase family genes *gp91*, *p22*, *p47*, *p67-Phox*. And upregulation of *ULK1*, *ATG14*, *BECN1*, and *LC3* Transcriptomics: mRNA expression downregulated of *ATP1A1* and *ATP5A1* Proteomics: Increase the phosphorylated ULK1, ATF4, Beclin-1, and LC3; Decrease the phosphorylation of BCl-2, MCl-1; Upregulation of LKB1, AMPK; Downregulation of Akt/mTOR/S6K Metabolomics: ROS levels were increased	s-µ*g*	3D clinostat	[118]
HL-60	Proteomics: Reduced expression of CD116 antigen Metabolomics: Reduced ROIS respiratory impulses	s-µ*g*	RWV	[253]
	Proteomics: Increase of IL-6, IL-8 and MCP-1 Metabolomics: Upregulation of nitric oxide	s-µ*g*	RCCS	[136]
	Genomics: Increase DNA damage Transcriptomics: Upregulation of transcript of *ATM*, *ATR*, *CHK1*, *CHK2*, and *RAD51*. Downregulation of transcript of *XPC*, *MLH1*, and *PMS2* Proteomics: Reduced expression of PCNA, ERK1/2AKK; Increase the phosphorylation of ATM; Upregulation of H2A.x, KU70, KU80, DNA-PKCs, Rad 51 Protein, Caspase-3, PARP, and Bax; Downregulation of Bcl-2 Metabolomics: Enhanced ROS formation	s-µ*g*	RCCS	[137]
UT-7/EPO	Transcriptomics: Decrease of mRNA expression of EPOR Proteomics: Activation of caspases-3 and downregulation of Bcl-xL	s-µ*g*	RCCS	[138]
SARCOMAS				
Hu09	Proteomics: alkaline phosphatase activity and osteocalcin production significantly reduced; 1,25-dihydroxy-vtamin D3-induced secretion of bone γ-carboxyglutamic acid-containing protein (BGP)	s-µ*g*, 7 d	Clinostat	[143]
A673	Transcriptomics: *CXCR4* and *CD44*: elevated in MCS; *DKK2*, and *VEGFA*; downregulated in AD and MCS; *EWS/FLI1* significantly upregulated in AD and MCS Proteomics: EWS/FLI1 protein was elevated in AD	s-µ*g*, 24 h	RPM	[144]
MG-63	Transcriptomics: *COL1A1* reduced Proteomics: osteocalcin, ALP, and vitamin receptor (VDR) protein levels: reduced	s-µ*g*, 3 d, 8 rpm and 16 rpm; microcarrier beads	RCCS/STLVs	[145]
	Transcriptomics: *COL1A1*, *BGLAP*, and *ALPL* reduced Proteomics: ALP activity following treatment at µ*g* increased 1.8-fold, compared to 3.8-fold at 1*g*; no change in collagen type I synthesis µ*g* vs. 1*g*	Foton 10 satellite, r-µ*g;* 9 d	spaceflight	[146]
	Transcriptomics: *VEGF121*, *VEGF165*: increased and *VEGF189*: decreased in both SiScr anSiRhoA cells; SiCdc42 cells only showed *VEGF165* upregulation Proteomics: In space fibrinogen was significantly reduced in all cell types except for SiRac1 cells	FOTON M3 satellite; r-µ*g*, 69 h	spaceflight	[148]
THYROID CANCER				
ML-1	Proteomics: collagen I, III, fibronectin, laminin, chondroitin sulfate, vimentin, TSH receptor Fas/Apo-1, 85-kDa PARP fragment and p53 increase, thyroglobulin decrease. Secretomics: reduced fT3, fT4 secretion.	s-µ*g*, 24 h	3D Clinostat	[150]
	Transcriptomics: 148 significantly regulated genes.	r-µ*g*, 31 × 22 s	PF	[151]
ML-1 WRO	Proteomics: β-actin, cytokine release and cytoskeletal protein expression.	s-µ*g*, 3 + 7 d	RPM, 2D Clinostat	[152]
WRO	Transcriptomics: *VEGFA*, *VEGFD*, *MSN*, and *MMP3* upregulation	s-µ*g*, 24 h	RPM	[153]
FTC-133	Proteomics: alpha-enolase, phosphoglycerate kinase 1, annexin 1 and 2 downregulation, glutathione S-transferase upregulation.	s-µ*g*, 72 h	RPM	[154]
	Transcriptomics: *CTGF*, *TLN1*, *IL6*, *CXCL8*, *CD44*, *SPP1, PCDHB5*, *PCDH7*, *CX3CL1*, *RAPGEF3*, and 13 ribosomal protein coding genes. Proteomics: RelA upregulation.	s-µ*g*, 24 h	RPM	[155]
	Transcriptomics: *EGF* and *CTGF.*	r-µ*g*, s-µ*g*, 10 d	Shenzhou 8, RPM	[156]
	Transcriptomics: *IL6*, *CXCL8*, *IL15*, *SSP1*, *VEGFA*, *VEGFD*, *FGF17*, *MMP2*, *MMP3*, *TIMP1*, *PRKAA*, and *PRKACA.*	r-µ*g*, s-µ*g*, 10 d	Shenzhou 8, RPM	[42]
	Transcriptomics: *CAV1* and *CTGF.*	s-µ*g*, 72 h	RPM, 2D Clinostat	[157]
	Proteomics: 47 and 13 unique proteins in ground control and flown cells, respectively.	r-µ*g*, 32 d	SpaceX CRS-3/ISS	[158]
	Proteomics: caveolin-1, plasminogen	r-µ*g*, 32 d	SpaceX CRS-3/ISS	[159]
	Exosomes: differences in the distribution of subpopulations and alteration of their population regarding the tetraspanin surface expression.	r-µ*g*, 32 d	SpaceX CRS-3/ISS	[160]
	Exosomes: altered relative quantification of 119 miRNAs.	r-µ*g*, 32 d	SpaceX CRS-3/ISS	[161]
	Transcriptomics: *VCL*, *PXN*, *ITGB1*, *RELA*, *ERK1*, and *ERK2* downregulation. Secretomics: angiopoetin-2.	r-µ*g*, 5 + 10 d	SpaceX CRS-13/ISS	[162]
	Transcriptomics: *NGAL*, *VEGFA*, *OPN*, *IL6*, and *IL17*. Secretomics: VEGFA, IL-17, and IL-6	s-µ*g*, 14 d	RPM	[163]
	Transcriptomics: *ACTB*, *TUBB1*, *VIM*, *LAMA*, *BAX*, *BCL2*, and *EGF* downregulation.	r-µ*g*, 6 min	TEXUS-53	[164]
	Transcriptomics: *COL1A1*, *VCL*, *CFL1*, *PTK2*, *IL6*, *CXCL8*, and *MMP14*.	18*g*, 1 min	MuSIC centrifuge	[165]
	Transcriptomics: reactions on dexamethasone treatment, NFκB components, genes of Wnt/β-catenin and TGF-β metabolic pathways.	s-µ*g*, 4 h + 3 d	RPM	[166]
FTC-133, WRO	Transcriptomics: Proteomics: reactions on dexamethasone treatment	s-µ*g*, 3 d	RPM	[167]
PROSTATE CANCER				
DU-145	Proteomics: cytokeratin 18, actin and vimentin increase	s-µ*g* 11 d	HARV	[169]
	Proteomics: ceramides, phospholipase, cAMP	s-µ*g*, 2–14 d	HARV	[174]
LNCaP	Proteomics: increase in PSA production under dihydrotesterone (DHT) treatment	s-µ*g*, 10 d	low-turning lateral vessel (STLV)	[170]
LNCaP, DU-145, PC-3	Proteomics: increased expression of E cadherin and CD44 in LNCaP	s-µ*g*, 21 d	HARV	[171]
PC-3	Transcriptomics: *VEGF*, *SRC1*, *AKT*, *MTOR* and *COL1A1* (downregulation), *ERK1/2*, *FN1*, *VCL1* (upregulation). Secretome: VEGF and NGAL (downregulation).	s-µ*g*, 3–5 d	RPM	[178]
	Transcriptomics: *ACTB*, *MSN*, *COL1A1*, *FN1*, *IL1A*, *IL6*, *CXCL8*, *LAMA3*, *TIMP1*, *FLT1*, *HIF1A* and *EGFR1* upregulation. Secretome: cytokines, TNF-α, collagen-1α1, MMP-2 and osteopontin (early) downregulation.	s-µ*g*, 0.5–24 h	RPM	[177]
	Transcriptomics: 298 gravisensitive genes, chemokines enriched	r-µ*g*, 31 × 22 s	PFC	[176]
BREAST CANCER				
MDA-MB-231	Proteomics: MCS: reduced cyclin-D1 protein content; Proapoptotic factors (BAX, PARP) increase in suspended RPM-cultured cells; prosurvival factors (Bcl-2, Survivin) are significantly decreased; p-AKT and p-ERK were significantly reduced in suspended cell aggregates	s-µ*g*, 24 h, 72 h	RPM	[186]
	Proteomics: Proteomic analysis of both EVs and cells further revealed a significant correlation with GTPases and proliferation	s-µ*g*, 96 h	Gravite^®^	[189]
	Transcriptomics: upregulation of ICAM1, CD44 and ERK1 mRNAs after the first parabola (P1) and a delayed upregulation of NFKB1, NFKBIA, NFKBIB, and FAK1 after the last parabola (P31). Proteomics: ICAM-1, VCAM-1 and CD44 protein levels were elevated, whereas the NFκB subunit p-65, annexin-A2 protein, and osteopontin protein levels were reduced after the 31st parabola (P31)	r-µ*g*; 31 parabolas	PFC	[192]
MCF-7	Transcriptomics: The *ACTB*, *TUBB*, *EZR*, *RDX*, *FN1*, *VEGFA*, *FLK1 Casp9*, *Casp3*, *PRKCA* mRNAs were downregulated in 5d-MCS-samples. *ESR1* was upregulated in AD, and *PGR1* in both phenotypes after 5 d. Proteomics: Increase in beta-actin, pan-cytokeratin protein in 5-day AD and MCS vs. 1*g*; Decrease in radixin in 5-day AD and MCS vs. 1*g*; Decrease in laminin and integrin-b1 in 5-day MCS vs. AD Elevation of fibronectin in MCS vs. AD; No change in VEFGA protein in 5-day study	s-µ*g*, up to 5 d	RPM	[95]
	Transcriptomics: Relative expression of *BCAR1*, *MAPK8*, and *CDH1* is downregulated in MCS Proteomics: Proteins in the AD and MCS group with LFQs deviated at least twofold compared with 1*g* control cells. E-cadherin was reduced in MCS cells, elevated proteins of the E-cadherin auto-degradation pathways. Higher concentration of BCAR1 and MAPK8 (JNK1) in AD than in MCS cells. The protein changes correspond rather well to the LFQ values as well as to the corresponding mRNA expression in MCS cells	s-µ*g*, 14 d	RPM	[187]
	Transcriptomics: early upregulation of *KRT8*, *RDX*, *TIMP1*, *CXCL8* mRNAs, and a downregulation of VCL after the first parabola of a parabolic flight Proteomics: Increase in VEGFA protein during the r-µ*g* phase (TEXUS), decrease in IL-6, IL-8 and MMP-9 proteins after the hyper-*g* phase (TEXUS); Significant downregulation of ITGB-1 protein and focal adhesion proteins after the first and 31^st^ parabolas: E-cadherin, vinculin; Increase in VEGFA and Il-8 after the first and 31st parabola	r-µ*g*	TEXUS sounding rocket flight, PFC	[191]
MCF-7 MDA-MB-231	Transcriptomics: MCS: *ERK1*, *AKT1*, *MAPK14*, *EGFR*, *CTNNA1*, *CTNNB1*, *ITGB1*, *COL4A5*, *ACTB*, and *TUBB* mRNAs differentially regulated	s-µ*g*, 14 d	RPM	[196]
CRL2351	Transcriptomics: AD, MCS: Upregulation of *VIM*, *RHOA*, *MAPK1*, and *BRCA1*; MCS: upregulation of *ERBB2; VEGFA* mRNA was unaltered Proteomics: Increase in vimentin and MAPK1 protein, no changes were detected for RHOA; and BRCA1 proteins in AD and MCS; reduced VEGF protein in AD and MCS	s-µ*g*, 5 d	RPM	[194]
GYNELOGIC CANCER				
CaSki	Transcriptomics: *SOX4*, *MALAT-1*, *COX4I1*, *C14orf45*, *ZNF12*, *HNRPH1*, *DENND1A*, *SMUG1*, *MELK*, *GPI*, *MMP7*, *STC1*, *JAGN1*, *STAU1*, *SUB1*, *ACTB*, *SHOC2*, *RBMS3*, *DIS3L2*, *SGEF*, *PDGFRL*, *NUCKS1*, *TP53BP1*, *AKR1B1*, *SMC1A*, *ARL6IP1*, *B2M*, *28. PSME1*, *CAPZA1*, *H2AFV*, *TBC1D20*, *NDUFA4*, *FTH1*, *GAPDH*	r-µ*g*	Shenzhou-4	[204]
OV-90, TOV-21G, and Caov-3	Proteomics: Downregulation of caveolin-1 protein	r-µ*g*	Clinostat	[205]
GASTROINTESTINAL TUMORS				
HCT116	Proteomics: ATG5 ATG12, FOXO3, PTEN upregulated and strong YAP nuclear localization in s-µ*g*	s-µ*g*	RCCS	[209]
MIP-101	Proteomics: lower expression of EGF-R, TGF-alpha, or TGF-beta in both r-µ*g* and s-µ*g*. greater CEA expression in r-µ*g*	r-µ*g* in low orbit, and s-µ*g*	RWV	[210]
DLD1	Genomics: *ARRDC3*, *ATF3*, *CCPG1*, *CDKN2AIP*, *CDKN2D*, *CREBBP*, *CREBRF*, *CXCL3*, *DDIT3*, *EGR2*, *ETS1*, *ETV5*, *FGF7*, *GORAB*, *HDAC9*, *HINT3*, *HIVEP2*, *IRS2*, *JUN*, *MIR1304*, *NCOA7*, *NDFIP2*, *PIBF1*, *PLEKHF2*, *PTEN*, *RAB30*, *SKIL*, *SMAD7*, *TNFAIP3*, *XIAP*, *ZFAND2A*, *ZFY*, *ZMYM5* Genes have been upregulated. *ASIC1*, *CD24*, *CDKN1C*, *DHFR*, *DNHD1*, *DUT*, *EEF1A1*, *EIF4A1*, *ENSA*, *FANCL*, *FAR1*, *FGFR3*, *GSPT1*, *GSTA4*, *HES4*, *HMGB1*, *HMGB3*, *HSPA4*, *IFI30*, *IFRD2*, *JPH1*, *NRBP2*, *MTPAP*, *NEURL1B*, *NFIA*, *PARP1*, *PHKA1*, *PLXNA1*, *PNPT1*, *POLR3H*, *RBBP4*, *TUBB* Genes have been downregulated. Transcriptomics: mRNA levels of cell cycle genes *CDK1*, *CDK2*, *CCNB1*, and *CCNE1* diminished in and Cell cycle inhibitors *CDKN2B* (p1INK4b) and *CDKN2D* (p16INK4d) were significantly higher in s-µ*g*. Proteomics: The protein expression of p38 MAPK, STAT3, E-Cadherin, PTEN, and MnSOD upregulated. The protein levels of AKT, as well as phosphorylated forms pAKTs473 and pAKTt308, the phosphorylated form of GSK-3β, diminished in s-µ*g*.	s-µ*g*	HARV	[111,211]
LS180	Genomics: The P-gp efflux pump gene expression in spheroids was increased after treatment with paclitaxel. Proteomics: intracellular ATP content increased, and AK decreased in s-µ*g*. Metabolimics: Glucose consumption increased	s-µ*g*	Clinostat	[212]
HGC-27	Metabolomics: Phosphatidyl choline, phosphatidyl ethanolamine, arachidonic acid, and sphinganine upregulated. Phosphatidyl serine, sphingomyelin, phosphatidic acid, creatine, L-proline, pantothenic acid, adenosine triphosphate, oxidized glutathione, and adenosine triphosphate downregulated.	s-µ*g*	RCCS	[213]
PaCa44	Transcriptomics: PI3k/Akt and NFκB signaling upregulated and eIF2 signaling downregulated after 9 days in s-µ*g*. Proteomics: Upregulation of stemness-related proteins, including EpCAM, ALDH1A3, ALDHA3A2, S100A4, ROHA, and ITGA3, glycolysis-related proteins logFC for ENO1, GAPDH, ALDOA, PGAM1, TPI1, and PKMI. Metabolomics: Glycolysis was upregulated and LDH downregulated after 7 and 9 days in s-µ*g*. Total TG levels decreased at 1 and 7 days but increased at 9 days. Total lysophosphatidylcholines (LPCs), lysophosphatidylethanolamine (LPEs), and polyunsaturated fatty acids (FAs) increased at 1 and 7 days but decreased at 9 days in s-µ*g*.	s-µ*g*	RPM	[215]
Caco-2	Proteomics: The proteins associated with ATP synthesis and mitochondrial functions MTFR2, MT-ATP8 were downregulated, and ubiquitin associated proteins UBAP1, UBR3 and cadherin 17 upregulated in s-µ*g*. Heterogeneous nuclear ribonucleoprotein D-like (HNRNPDL), specifically its subunit DnaJ homolog subfamily C member 5 and CDK2 were downregulated	s-µ*g*	2D clinostat	[216]
HepG2	Genomics: serine hydroxymethyltransferase 2, insulin receptor, apolipoprotein E, cyclophilin F, as KIAA0073 protein, EST (Hs.55153) upregulated. *CYP1A1*, *AKR1C1*, *EPHX1*, *LTB4DH*, *LDLR*, *HMGCR* Metabolic genes were upregulated. Metabolomics: Genes responsible for lipid transporter activities, such as *APOA1*, *APOA2*, and *APOB*, were significantly downregulated.	s-µ*g*	RCCS, RWV	[217,218,219]
EPG85–257 RDB, EPG85–257 P	Genomics: *ABCB1* gene in EPG85-257 RDB cells downregulated in presence and absence of DOX in s-µ*g*. *ABCB1* and *ABCG2* genes downregulated in EPG85–257 P in presence of DOX in s-µ*g*.	s-µ*g*	RCCS	[214]
LUNG CANCER				
A549	Transcriptomics: *MMP2*, *MKI67* mRNAs reduced in s-µ*g* (ns).	s-µ*g*, 72 h	clinostat	[222]
	Transcriptomics: differentially expressed miRNAs: cell cycle (via P53, CDKN, E2F), apoptosis (via BCL2, BIRC5), and stress response (via FOS, MAPK).	s-µ*g*, 48 h	RPM	[225]
	Transcriptomics: two datasets from the Gene Gene Expression Omnibus (GEO) database (GSE78210 and GSE36931: gene expression profiles for A549 and Colo699) Candidate genes: *AZGP1*, *CFB*, *NOX1*, *VTCN1*, *AGR3*, *GDA*, *TCN1*, *CST1*, *F5*, *CEACAM6*, *BPIFB1*, *FCGBP*, and *BPIFA1*. Increase in *CDH1*, significant decreases in *CDH2* and *MMP2*—induction of mesenchymal–epithelial transition	s-µ*g*, 24 h, 48 h, 72 h	RWV	[226]
A549 and H1703	Transcriptomics: *MMP2*, *MMP9*, *TIMP1*, and *TIMP2* revealed a significant downregulation of all genes in A549 cells under s-µ*g*. In H1703 cells, *MMP2*, *MMP9:* significantly downregulated Proteomics: MMP-2, MMP-9, and TIMP-1: increase after 24 h; decrease after 36 h of s-µ*g*	s-µ*g*, 36 h	3D clinostat	[223]
CRL-5889	Transcriptomics: AD cells showed differentially increased expression of genes involved in cell death/apoptosis, such as *TP53*, *CDKN2A*, *PTEN*, and *RB1*.	s-µ*g*; max. 96 h	RPM	[227]

Abbreviations: min, minute(s); h, hour(s); d, day(s); r-µ*g*, real microgravity; s-µ*g*, simulated microgravity; 2D, two-dimensional; 3D, three-dimensional; DLR, Deutsches Zentrum für Luft- und Raumfahrt, engl. German Aerospace Center; ISS, International Space Station; RVW, Rotating Wall Vessel; RPM, Random Positioning Machine; RCCS, Rotating Cell Culture System; PFC, Parabolic Flight Campaign; STS, Space Transportation System TEXUS, Technologische Experimente unter Schwerelosigkeit, engl. Technological Experiments in Zero Gravity; HARV, High Aspect Ratio Vessels; STLV, low-turning lateral vessel; max., maximum; MuSIC, Multi Sample Incubator Centrifuge; SpaceX CRS-13, also known as SpX-13, Commercial Resupply Service mission; Gravite^®^ is a 3D-clinostat, a multi-directional gravity device for simulating microgravity.
